# Dietary Supplementation With ω6 LC-PUFA-Rich Algae Modulates Zebrafish Immune Function and Improves Resistance to Streptococcal Infection

**DOI:** 10.3389/fimmu.2018.01960

**Published:** 2018-09-06

**Authors:** Sagar Nayak, Inna Khozin-Goldberg, Guy Cohen, Dina Zilberg

**Affiliations:** ^1^The French Associates Institute for Agriculture and Biotechnology for Drylands, The Jacob Blaustein Institutes for Desert Research, Ben-Gurion University of the Negev, Beersheba, Israel; ^2^The Skin Research Institute, Dead Sea and Arava Science Center, Masada, Israel

**Keywords:** arachidonic acid, dihomo-γ-linolenic acid, disease resistance, immune function, LC-PUFA, microalgae, fish

## Abstract

Arachidonic acid (ARA, 20:4*n*-6) and dihomo-γ-linolenic acid (DGLA, 20:3*n*-6) are omega-6 long-chain polyunsaturated fatty acids (LC-PUFA), which are key precursors for lipid mediators of the immune system and inflammatory response. The microalga *Lobosphaera incisa* (WT) and its Δ5-desaturase mutant P127 (MUT) are unique photosynthetic sources for ARA and DGLA, respectively. This study explores the effect of dietary supplementation with *L. incisa* and P127 biomass on tissue fatty acid composition, immune function, and disease resistance in zebrafish (*Danio rerio*). The broken microalgal biomass was added to commercial fish feed at 7.5 and 15% (w/w), providing 21.8 mg/g feed ARA for the WT-supplemented group and 13.6 mg/g feed DGLA for the MUT-supplemented group at the 15% inclusion levels. An unsupplemented group was used as the control. After 1 month of feeding, fish were challenged with *Streptococcus iniae*. Fish were sampled before the challenge and 1 week after the challenge for various analyses. Tissue ARA and DGLA levels significantly increased in the liver, corresponding to microalgal supplementation levels. The elevated expression of specific immune-related genes was evident in the kidneys in all treatment groups after 1 month of feeding, including genes related to eicosanoid synthesis, lysozyme, and NF-κB. In the liver, microalgal supplementation led to the upregulation of genes related to immune function and antioxidant defense while the expression of examined genes involved in ARA metabolism was downregulated. Importantly, fish fed with 15% of both WT- and MUT-supplemented feed showed significantly (*p* < 0.05) higher survival percentages (78 and 68%, respectively, as compared to only 46% in the control group). The elevated expression of genes related to inflammatory and immune responses was evident post-challenge. Collectively, the results of the current study demonstrate the potential of microalgae-derived dietary ARA and DGLA in improving immune competence and resistance to bacterial infection in zebrafish as a model organism.

## Introduction

Alterations in the dietary composition and proportion of omega-3 and omega-6 long-chain polyunsaturated fatty acids (LC-PUFA) groups are known to affect a wide range of physiological processes (1, 2). In addition to their structural and metabolic functions, omega-6 (*n*-6) LC-PUFA are precursor molecules for numerous bioactive lipid mediators. These may regulate and impact immune function and inflammatory responses, exerting both pro-inflammatory and anti-inflammatory effects (3), depending on the specific PUFA precursor, environmental stimuli and the enzymatic pathway activated. Arachidonic acid (ARA; 20:4*n*-6) and dihomo- γ-linolenic acid (DGLA; 20:3*n*-6) are precursors for different types of bioactive eicosanoids. Cyclooxygenases (COX) initiate the conversion of ARA and DGLA to prostaglandins, among them prostaglandin series 1 and series 2 (e.g., PGE_1_ and PGE_2_), respectively (4). Primarily the series-1 prostaglandins are viewed as anti-inflammatory, while the series-2 are known for their pro-inflammatory action (4, 5). However, there are a few exceptions to these predominant roles; thus, their impact on modulating the immune system seems to be more complex (6). The effects of lipoxygenase (LOX) and CYP450-mediated activities on ARA and DGLA lead to the formation of a diverse range of lipid mediator molecules, whose mode of action is dependent on the LC-PUFA substrate. The series-2 eicosanoids generated from ARA are the most studied. Arachidonic acid and its metabolites are well known for their roles in immunity and inflammation in mammals (7, 8). Recent studies have demonstrated the importance of ARA nutrition in fish and its direct effects on growth, survival, fatty acid profile, immune responses, lipid metabolism, and reproduction (9–16). In the case of DGLA, studies with mice and mammalian models have revealed DGLA's multiple anti-inflammatory activities, including the prevention of platelet aggregation, hypertension and allergies (17–21). However, there are no reports of studies on DGLA dietary supplementation in fish, largely due to limitations in the availability of this rare LC-PUFA. In this context, the microalga *Lobosphaera incisa* wild type (WT) and its Δ5-desaturase mutant P127 (MUT) are unique photosynthetic sources for ARA and DGLA, respectively (22, 23), whose potential in aquaculture nutrition is yet to be explored. Microalgae are an emerging resource for biochemical diversity and various health-promoting ingredients, such as LC-PUFA, carotenoids, exopolysaccharides and more. Microalgae are a rich source of *n*-3 LC-PUFA, while accumulation of *n*-6 LC-PUFA is a rare phenomenon (24). Microalgae offer health benefits as a nutritional supplement in aquaculture feeds because of their digestibility and high content of proteins, lipids, and essential nutrients (25). Aquaculture nutrition requires alternative sources of sustainable dietary protein and LC-PUFA to replace currently diminishing resources (26). Microalgal biotechnology can offer a renewable supplementation means, compared to the traditional fish-based LC-PUFA sources (27). While the majority of research efforts have addressed *n*-3 LC-PUFA supplementation, known for their health-promoting benefits, in our research, we focus on the *n*-6 LC-PUFA-accumulating microalga *L. incisa*.

Diseases are a major cause of morbidity and mortality in commercial aquaculture, leading to fish loss and associated economic impacts. Increasing fish's resistance to infectious agents would aid in reducing the impact of pathogen occurrence. The present study aimed at evaluating the effects of dietary supplementation with microalgal biomass enriched in ARA and DGLA, using the zebrafish. We examined the effects of dietary supplementation on fatty acid (FA) composition, expression of immune and inflammatory genes, and resistance to infection with *Streptococcus iniae*. Furthermore, fish surviving the *S. iniae* challenge were analyzed to understand the role of modified diets in modulating immune gene expression and FA composition during infection. Selected doses of microalgae for dietary supplementation were based on previous studies that were conducted in our laboratory, showing positive results at supplementation rates similar to those selected in this study (10, 11, 28). Results from this study showed that dietary microalgal supplementation altered the ARA/DGLA levels in tissues and played a key role in increasing resistance to infection and in modulating host gene expression.

## Materials and methods

### Fish husbandry

Wild type zebrafish (*Danio rerio*) were raised in the lab and maintained at a temperature of 28°C with a light:dark cycle of 12:12 h. Prior to the start of the trial, fish were fed at 2% of their body weight once a day with a commercial feed (Ocean Nutrition, San Diego, USA). During the entire experimental period, tanks were siphoned every other day, and 10% of water exchange was carried out. Temperature was monitored daily, and measurements of water quality parameters including ammonia and nitrite were monitored twice a week using colorimetric test kits (Merck, Germany). Ammonia (0–0.2 mgL^−1^), nitrite (0–0.025 mg L^−1^), nitrate (0–10 mg L^−1^), and oxygen were maintained at over 80% saturation. The experimental trial was approved by the Ben-Gurion University Committee for the Ethical Care and Use of Animals in Experiments (authorization no. IL 58-09-2016).

### Microalgal production and preparation of experimental diets

The microalga *Lobosphaera incisa* WT and its Δ5-desaturase mutant P127 were used as sources of ARA and DGLA, respectively. The algae were cultivated in 1-L glass columns in a nitrogen-depleted BG-11 medium to induce accumulation of the major respective LC-PUFA, as previously described (11), and were harvested after 14 days of cultivation. A broken cell algal powder was prepared as described in Dagar et al. (11). Microalgal biomass was added to the commercial fish feed (Ocean Nutrition, San Diego, USA) at 7.5 and 15% (w/w) for preparation of experimental diets. In brief, powdered commercial zebrafish feed and algal powder were mixed by adding cold double-distilled water (DDW) until a doughy texture was obtained. The mixture was then spread on a tray and freeze-dried in a lyophilizer. The dried mixture was broken and passed through a mesh sieve of 500 microns to obtain final sizes and concentrations of (6.8 and 13.7 mg DGLA/g feed) and (10.8 and 21.8 mg ARA/g feed). A similar method was followed for preparing the control feed without the addition of algal biomass. The experimental feeds were analyzed for fatty acid composition and content by gas chromatography. The total carbon and nitrogen content in the experimental feed was determined using an automated elemental analyzer (Thermo, USA). The total nitrogen content was multiplied by a conversion factor of 6.25 (equivalent to 0.16 g nitrogen per gram of protein) to estimate the amount of protein in the sample.

### Dietary feeding experiment and sampling

The dietary trial was designed to test five different treatments comprising two supplementation levels of two types of dried broken algae and, consequently, of ARA and DGLA, and one group of an unsupplemented control. Similarly sized adult zebrafish (ca. 5–6 months old), originating from a single cohort, were distributed among 20 aquaria, 30-L each (30 fish/tank), supplied with individual submerged biological filters and aeration. Each of the five treatments was tested in four replicates. Fish were fed with experimental and control diets for a period of 4 weeks at 2% body weight per day, in two separate feed applications. Fish weight was determined at the beginning of the trial (total of 20 fish) and then again after the 4 weeks of feeding, by weighing 20 fish from each treatment group. At the end of this period, 20 fish from each aquarium were infected with *S. iniae* at an LD_50_ dose of 10 CFU per fish (see details on *S. inae* preparation below) by an intraperitoneal (IP) injection of 10 μl, using a high precision syringe pump-Fusion 720 (Chemyx, Stafford, USA). Mortality was monitored daily for a period of 1 week. Fish were sampled at the completion of 4 weeks of dietary supplementation for determining FA composition, PGE_2_ production and gene expression analysis. For each experimental treatment, 20 fish were sampled for gene expression analysis (liver and kidneys), and another 20 fish were sampled for determining FA composition and prostaglandin production. Organs were weighed at sampling using analytical scales. Each sample consisted of a pool of five fish from every tank, with four pools per treatment. All sampled fish were euthanized in clove oil (250 ppm) prior to dissection. Liver and kidney samples collected for gene expression analysis were immediately placed in RNA later™ (100 mg/500 μl) at room temperature and left for 6 h at 4°C before transferring to −80°C for storage. Liver and kidney samples to be analyzed for FA composition and prostaglandin production, respectively, were collected in 1.5-ml tubes on dry ice, and immediately snap-frozen in liquid nitrogen and stored at −80°C until analysis. A similar method of sampling and sample processing for gene expression, FA composition and PGE_2_ production was repeated for surviving fish after completing 1 week of the *S. inae* challenge.

### *Streptococcus iniae* challenge

After 1 month of dietary supplementation, the fish were challenged by an IP injection of a 10-μl inoculum of *S. iniae* in a 10^3^ cfu ml^−1^ cell suspension. This dose was pre-calibrated and confirmed to induce 50% mortality in the zebrafish (LD_50_, data not shown). The inoculum was prepared by inoculating frozen bacterial stock (from −80°C) into fresh TSB broth in a 15-ml tube. The cultures were grown at 25°C to the mid-logarithmic phase of growth (until OD 600 nm reached 0.250, corresponding to 10^8^ cells ml ^−1^). One milliliter of this culture was centrifuged at 1500 × *g* for 10 min at 4°C and washed in sterile phosphate buffered saline (PBS). Serial dilutions were carried out from this tube to achieve a final inoculum concentration of 10^3^ cell ml ^−1^. The final cell concentration was confirmed by subsequent plating of the inoculum on TSB plates using the drop plate method.

### Quantitative PCR analysis

Frozen tissue samples were homogenized in a mixer mill (Retsch, Germany) using metal beads with continuous cooling using liquid nitrogen. RNA extraction from tissue homogenates was carried out using a SV Total RNA Isolation Kit (Promega, USA) based on the manufacturer's instructions. Total RNA was eluted and quantified on a Nanodrop spectrophotometer (Thermo, USA) and reverse transcribed using the Verso cDNA Synthesis Kit (Thermo Scientific, USA) following the manufacturer's instructions. The resulting cDNA was used as a template for quantitative PCR in a Biorad CFX96™ real-time PCR detection system with SsoAdvanced™ Universal SYBR Green Supermix (Biorad, USA). Primers used in this study are shown in Table 1. Three technical replicates from each of the four biological replicates were used in the qPCR assay. The reaction mixture consisted of 5 μl of SYBR green master mix, 0.5 μl (250 nM) each of forward and reverse primer gene specific primers and 4 μl of diluted template cDNA. The PCR was then performed under the following conditions: initial denaturation at 95°C for 30 s followed by 40 cycles of 95°C for 15 s and 60°C for 15 s. The amplification efficiency of the primer pairs was assessed by generating standard curves and looking for linear amplifications of target genes from serial dilutions of the cDNA pool. The expression of the target genes analyzed were normalized to the expression of the elongation factor 1α gene (EF1). The *EF1* was chosen as the internal control after confirming its stable expression within all treatments (29). A melt curve analysis was carried out to demonstrate primer specificity and amplification of a single product. Expression values for target genes were normalized to *EF1*α using the formula 2^(−ΔCt)^, where ΔCt = avg. of target gene Ct—avg. of EF1 gene Ct.

**Table 1:** Primers used in this study for quantitative real-time PCR.

**Gene**	**Sequence 5′-3′**	**Accession no**.
Delta-6 fatty acyl desaturase	F-CATCACGCTAAACCCAACA	AF309556
	R-GGGAGGACCAATGAAGAAGA	
Phospholipase C γ1	F-ACCAGTTTTCCAGCGAGTCGT	NM_194407.1
	R-AGCAGTCCAACTCGATGCATC	
Cytosolic phospholipase A2	F-TGCTCTTGGAAGTTTGCGC	NM_131295.1
	R-TCTGCGTGTCTGCATGAACAG	
Cyclooxygenase-1	F-AAGGTCTGGGTCACGGAGTG	NM_153656.1
	R-CAAGAGAATCTCCATAAATGTGTCCA	
Cyclooxygenase-2	F-GTTAAAAGATGGAAAGCTTAAATACCAGG	NM_153657.1
	R-GGGTACACCTCACCATCCACA	
Arachidonate 15- lipoxygenase	F-ATGGAGCACTGGAAAGAAGACT	NP_955912.1
	R-TACAGTAAGCAGAGTGGGGCA	
Arachidonate 5-lipoxygenase	F-ATGGAGCACTGGAAAGAAGACT	NP_001290191.1
	R-TCCAGTGTGAGACCCCTCTC	
Interleukin 1β	F-CATTTGCAGGCCGTCACA	AY340959.1
	R-GGACATGCTGAAGCGCACTT	
Tumor necrosis factor	F-CCATGCAGTGATGCGCTTT	AY427649.1
	R-TTGAGCGGATTGCACTGAAA	
Toll-like receptor 22	F-CCAGCTCTCGCCGTACCA	AY389460.1
	R-TTGGGCCAGCGGATGT	
Complement C3b	F-CAGTGGGAATATGTTGGCATTG	AF047414
	R-TTAGCTGCCCTTCATAACCTGTT	
Lysozyme	F-AGGCTGGCAGTGGTGTTTTT	NM_139180.1
	R-CACAGCGTCCCAGTGTCTTG	
NF-kB	F-CGCAAGTCCTACCCACAAGT	NM_001353873.1
	R-ACCAGACTGTGAGCGTGAAG	
Glutathione peroxidase 3	F-TCCAGGAAATGGATTCGTTC	NM_001137555.1
	R-TCTCTCCTACAGGCGGACAT	
Catalase	F-AGGGCAACTGGGATCTTACA	AF170069
	R-TTTATGGGACCAGACCTTGG	
Superoxide dismutase	F-GGCCAACCGATAGTGTTAGA	Y12236
	R-CCAGCGTTGCCAGTTTTTAG	
Beta-actin	F-CGAGCAGGAGATGGGAAC	AF057040
	R-CAACGGAAACGCTCATTGC	
Elongation factor 1-alpha	F-CTTCTCAGGCTGACTGTGC	AY422992
	R-CCGCTAGCATTACCCTCC	

### Fatty acid analysis

Liver samples used for fatty acid analyses were kept frozen at −80°C and then lyophilized overnight. Total lipids were extracted from the powdered samples with the method of Bligh and Dyer (30) and evaporated to dryness under N_2_ flow. Separation of total lipid extracts into neutral and polar lipid fractions was performed by sequential elution with chloroform and methanol on silica gel cartridges (Bond- Elut, USA). Polar and neutral fractions were then transmethylated, and fatty acid methyl esters (FAMEs) were extracted as described in Nayak et al. (31). A GC analysis of FAMEs was carried out on a Trace Ultra gas chromatograph (Thermo Fisher Scientific, USA) equipped with a flame ionization detector, a programmable temperature vaporization injector and a capillary column (SupelcoWAX, Sigma-Aldrich, USA) as described in Nath et al. (14).

### Measurement of PGE_2_ by ELISA

Kidney samples were solubilized in 500 μl of ice cold acidified DDW (pH = 3.5), mixed vigorously, and homogenized on ice. The supernatant was collected and 100 μl aliquots were mounted on C18 flash columns (Sigma Aldrich, USA), under reduced oxygen conditions (N_2_:CO_2_, 95:5), to avoid sample oxidation. The columns were washed by ice-cold water, 15% ethanol and hexane, and the PGE_2_-containing fraction was eluted in ethyl acetate. The samples were dried under reduced oxygen conditions and re-dissolved in 500 μl of assay buffer. PGE_2_ was quantified by a PGE_2_ EIA kit (Enzo Life Sciences, US).

### Histology

For the histopathological analysis, whole fish were euthanized in clove oil (250 ppm) and fixed in 10% neutral buffered formalin. Fixed whole fish were cut into ca. 0.4-cm-thick transverse sections and processed using routine histological techniques as described in Sharon et al. (32). The slides were stained with H&E and examined under a light microscope. A general analysis was conducted, examining internal organs, heart, gills, and brain. To analyze differences, scoring was applied of 0, 1, and 2:0 for minimal liver vacuolization and 2 for high liver vacuolization.

### Statistics

The results of gene expression, relative FA compositions and histological analyses between experimental groups were compared by a one-way analysis of variance (ANOVA), using the Sigma Plot 13 software (Systat Software Inc., USA). Pairwise multiple comparisons were carried out using the Dunn's *post hoc* test (*p*-values < 0.05 were considered significant). The significance between gene expression values before and after challenge was determined using the *t*-test. Survival curves were generated by the Kaplan Meyers Survival Analysis and treatments compared using the Holm-Sidak method (Sigma Plot 13). A principal component analysis (PCA) was carried out using the FactoMineR package, and plots for the PCA were generated using ggplot 2 package in R studio version 1.14.

## Results

### Fatty acid composition of experimental diets

The FA composition of modified zebrafish feed reflected the supplemented microalgal LC-PUFA content (Table 3). Supplementation with broken *L. inicisa* WT and MUT significantly increased the content of ARA and DGLA in the supplemented diets, respectively, as compared to the control. Feed supplemented with 7.5 and 15% of WT contained elevated ARA levels, of 11.6 and 21.8 μg mg^−1^, respectively, compared to 0.6 μg mg^−1^ in the control. The amount of DGLA in these diets was similar to the level in the control, 0.3 and 0.6 μg mg^−1^ feed, respectively (Table 3). Diets supplemented with MUT contained elevated levels of DGLA, 7.6 and 13.4 μg mg^−1^ feed in the 7.5 and 15% supplemented diets, respectively, as compared to 0.1 μg mg^−1^ in the control. The ARA content was similar to that in the control (0.6 μg mg^−1^ in both diets). Total fatty acid content in the control feed was lower (76.1 μg mg^−1^) than in the supplemented feeds, in which levels ranged from 96–116.5 μg mg^−1^, indicating an increased fraction of microalgal FA. In line with the higher proportion of oleic acid (OA, 18:1*n*-9) in P127 biomass (Table 3), the MUT-supplemented feed contained higher proportions of 18:1*n*-9 (23 and 27% of TFA in the 7.5 and 15% supplementation, respectively), compared to the WT-supplemented and control feeds (15.7–16.3 % of TFA). In the WT-supplemented diets, the marked increase was solely in ARA levels, due to elevated ARA levels in the WT (Table 3). Enrichment with the biomass of both microalgae reduced the relative percentage of the *n-*3 fatty acids, eicosapentaenoic acid (EPA; 20:5*n*-3) and docosahexaenoic acid (DHA, 22:6*n*-3), in the experimental diets by up to 42% as compared to the control. It is worth noting that the supplementation did not affect the protein and carbon content in the diets (Table 3), which indicates that some fraction of protein was replaced by microalgal protein.

### Effects of dietary supplementation on fish and organ weight and on fatty acid composition in liver

Weight measurement revealed no significant difference between treatment groups at experiment termination (Table 2), and there was no evident growth during the trial, as the weight at time zero was similar to the fish weight after 4 weeks (average ranging between 0.27 and 0.28 g). Similarly, liver and kidney weight did not differ between treatment groups (Table 2).

**Table 2:** Average fish body and internal organ weights ± SD after 4 weeks of feeding with experimental diets (*n* = 20 per treatment).

**Treatment**	**Body weight (mg)**	**Liver weight (mg)**	**Kidney weight (mg)**
WT 7.5%	275 ± 30	6.7 ± 2.5	10.9 ± 5.4
WT 15%	280 ± 50	8.0 ± 3.2	13.0 ± 9.2
MUT 7.5%	271 ± 20	5.3 ± 1.3	10.6 ± 3.2
MUT 15%	283 ± 30	6.7 ± 3.1	16.0 ± 8.1
CONTROL	272 ± 30	9.0 ± 3.3	8.2 ± 1.6

**Table 3:** Fatty acid composition and content of commercial zebrafish feed (used as control) and feed supplemented (w/w) with different levels of microalgal biomass: *Lobosphaera incisa* WT and its Δ5-desaturase mutant P127 (MUT) at two levels (7.5 and 15% of feed).

	**Algae**		**Fatty acid composition (% of TFA) of experimental diets**				
	**WT**	**MUT**	**WT (ARA enriched)**		**MUT (DGLA enriched)**		**Unsupplemented**
Fatty acid			7.5%	15%	7.5%	15%	Control
14:0			2.8	2.2	2.6	2.0	3.4
16:0	10.3	8.0	16.9	15.2	15.6	14.1	18.4
16:1	0.0	0.0	3.4	2.8	3.2	2.6	4.1
16:2	0.1	0.1	0.3	0.2	0.3	0.3	0.3
16:3	0.7	0.0	0.4	0.5	0.4	0.4	0.3
18:0	2.2	4.8	3.0	2.8	2.8	2.6	3.2
18:1*n-*9	14.1	38.6	16.1	15.7	22.9	27.0	16.3
18:1*n-*7	3.9	2.4	3.0	3.1	2.7	2.7	2.6
18:2*n-*6	9.4	13.9	16.8	15.0	16.9	16.2	18.0
18:3*n-*6	0.8	1.2	0.2	0.3	0.3	0.5	0.1
18:3*n-*3	0.9	1.0	1.2	1.9	2.1	1.9	2.5
18:4	0.0	0.0	1.1	0.9	1.1	0.8	1.4
20:0	0.2	0.3	0.2	0.2	0.3	0.3	0.3
20:1	0.3	0.6	3.4	2.8	3.3	2.6	4.3
20:2	0.3	0.2	0.4	0.4	0.3	0.3	0.4
20:3*n-*6 (DGLA)	1.2	27	0.3	0.6	7.1	11.5	0.2
20:4*n-*6 (ARA)	52.8	0.1	12.1	20.1	0.6	0.6	0.8
20:3*n-*3	0.2	0.0	0.3	0.3	0.3	0.3	0.4
20:4*n-*3	0.0	0.0	0.5	0.5	0.6	0.6	0.7
20:5*n-*3 (EPA)	1.5	0.0	5.0	4.2	4.5	3.5	6.1
22:1	–	–	4.1	3.4	3.9	3.0	5.2
22:4*n-*6	–	–	0.1	0.1	0.1	0.1	0.1
22:5*n-*3	–	–	0.6	0.5	0.7	0.4	0.7
22:6*n-*3	–	–	7.1	5.6	6.6	5.1	8.8
**FA content (**μ**g mg**^−1^ DW)^*^							
TFA	274.9	337.5	95.9	108.2	106.5	116.5	76.1
ARA	145.1	0.2	11.6	21.8	0.6	0.6	0.6
DGLA	3.4	91.2	0.3	0.6	7.6	13.4	0.1
EPA	2.2	0.0	4.8	4.6	4.8	4.1	4.7
DHA	–	–	6.8	6.1	7.0	5.9	6.7
Protein (%)			59.2 ± 0.6	54.2 ± 0.5	58.0 ± 1.2	56.3 ± 1.0	60 ± 0.3
Carbon (%)			45.4 ± 0.5	54.28 ± 0.5	58.04 ± 1.2	56.37 ± 1.0	60 ± 0.3

*Values are presented as % of TFA, absolute content presented as μg mg^−1^ dry weight of feed*.

* *FA content represents μg of the FA per mg of dry food weight (DW)*.

**Figure 1:**
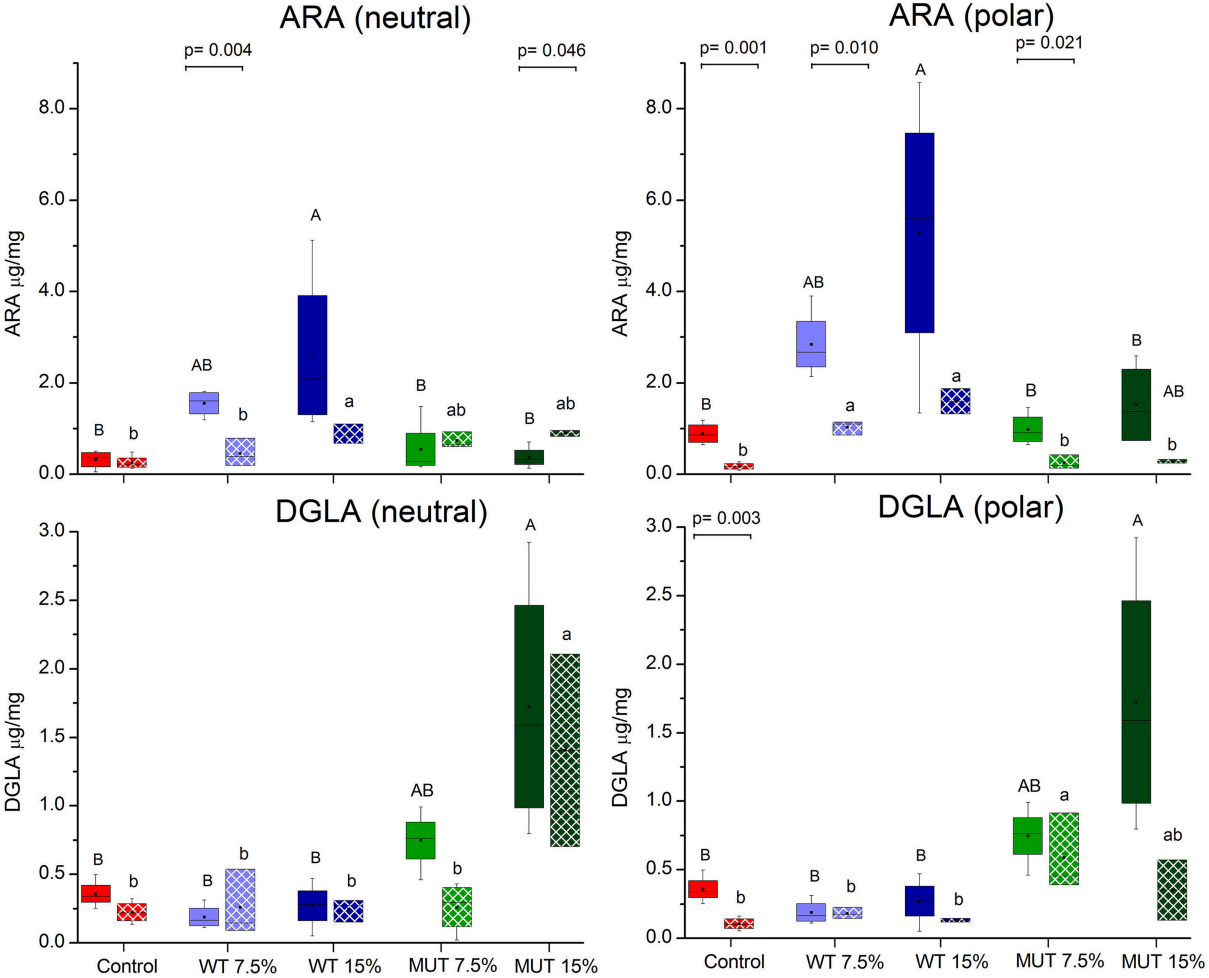
Changes in the content of ARA and DGLA in polar and neutral lipids of zebrafish liver before and after challenge. Box plot represents absolute content (ARA/DGLA μg/mg dry weight) ± SD (biological replicates = 4, *n* = 20). Fatty acid content before and after challenge is represented by solid colored and pattern-filled boxes, respectively. Significant difference between treatments before and after challenge is indicated in bracket with corresponding *p* value, as determined by *t*-test. A and B denote significant differences between treatments before challenge; a and b denote significant differences between treatments after challenge. Differences were considered significant at *p* < 0.05.

A fatty acid analysis was carried out on liver samples from zebrafish fed with the differently supplemented diets after 1 month of feeding. Both polar and neutral lipid fractions were analyzed. The main fatty acids constituting zebrafish liver lipids were palmitic acid (PA; 16:0), oleic acid (OA; 18:1*n*-9), and linoleic acid (LA; 18:2*n-*6), accounting for about 65% of neutral lipid and about 44% of polar lipid fatty acids. Among the highly unsaturated fatty acids (ω3 HUFA), DHA was present at a higher proportion in the polar lipid fraction in all treatment groups (ranging from 16.3 to 19.6% of TFA) as compared to DHA levels in the neutral lipids. The proportion of EPA in both polar and neutral fractions was <5% of TFA. Dietary supplementation with WT and MUT resulted in a significant increase in the proportion of ARA and DGLA, as a % of TFA, in both the polar and neutral lipid fractions of fish liver as compared to the control (Tables 4, 5). The increase was more prominent in the polar lipids (Table 5) and corresponded to the dietary supplementation levels. In the polar lipid fraction, ARA levels reached 8.8 and 11.3% of TFA in fish fed with 7.5 and 15% WT-supplemented diets, respectively, as compared to 2.2% in the control. In addition, the 22:4*n*-6 levels, an elongation product of ARA, were increased in the WT-supplemented fish. A significant decrease in the proportion of EPA was recorded in fish fed with the WT-supplemented feed as compared to fish fed with control feed. Similarly, an increase in the proportion of DGLA was observed in fish fed with the MUT-supplemented feed, in which DGLA levels reached 2.6 and 4% of fatty acid content in the polar lipid fraction of 7.5 and 15% supplemented groups, respectively, as compared to only 0.9% in the fish fed with control feed (Table 5). Changes in the fatty acid composition of neutral lipids were also significant, but less prominent (Table 4). Due to *L. incisa* supplementation, ratios of total ω3 to total ω6 HUFA decreased considerably in all treatment groups as compared to groups fed with control feed. A significant increase in the ARA and DGLA content of dry weight (μg/mg) was observed in the liver of zebrafish, corresponding to the dietary supplementary levels (Figure 1). The incorporation was more evident in the polar lipid fraction than in the neutral lipid fraction.

**Table 4:** Fatty acid composition (% of TFA) and content in liver neutral lipids of zebrafish fed with a diet supplemented (w/w) with different levels (7.5 and 15%) of the microalga *Lobosphaera incisa* WT and mutant P127.

**Fatty acid**	**Before challenge**					**After challenge**				
	**WT 7.5 %**	**WT 15 %**	**MUT 7.5%**	**MUT 15%**	**Control**	**WT 7.5 %**	**WT 15 %**	**MUT 7.5%**	**MUT 15%**	**Control**
14:0	1.8 ± 0.3	1.9 ± 0.2	1.8 ± 0.1	1.8 ± 0.1	1.8 ± 0.2	2.1 ± 0.1	1.2 ± 0.8	1.4 ± 0.9	2.3 ± 1.1	1.8 ± 0.4
16:0	22 ± 1.3	21.4 ± 0.2	20.7 ± 0.5	20.4 ± 0.8	22.6 ± 1.0	30.2 ± 1.8	24.9 ± 3.1	23.8 ± 0.1	21.2 ± 3.8	24.4 ± 2.6
16:1*n*-5	0.8 ± 0.1	0.7 ± 0.1	0.9 ± 0.3	0.7 ± 0.1	0.8 ± 0.2	0.3 ± 0.5	0.4 ± 0.4	0.5 ± 0.4	0.6 ± 1.6	1.3 ± 2.1
16:1*n*-7	3.6 ± 0.4	3.6 ± 0.5	3.2 ± 0.2	3.2 ± 0.2	3.9 ± 0.2	2.9 ± 2.5	2.6 ± 1.9	2.2 ± 1.4	3.7 ± 0.4	2.9 ± 2.7
16:2	0.3 ± 0.1	0.3 ± 0.1	0.3 ± 0.2	0.3 ± 0.1	0.3 ± 0.2	0.4 ± 0	0.4 ± 0.4	0.4 ± 0.2	0.5 ± 0	0.3 ± 0.3
16:2	0.5 ± 0.2	0.5 ± 0.2	0.8 ± 0.1	0.5 ± 0.4	0.5 ± 0.1	2.5 ± 2.6	0.5 ± 0.2	2.7 ± 4.1	0.6 ± 0.7	1.7 ± 1.1
16:3	0.7 ± 0.3	0.7 ± 0.1	0.6 ± 0.1	0.5 ± 0	0.6 ± 0.1	0.6 ± 0.3	0.8 ± 0.6	0.6 ± 0.4	0.6 ± 0.3	1.0 ± 0.7
18:0	4.3 ± 0.4	4.2 ± 0.1	4.8 ± 0.8	4.5 ± 0.4	4.4 ± 0.2	9.1 ± 1.4	8.7 ± 1.8	13.0 ± 11.1	4.1 ± 7.1	10.6 ± 7.2
18:1*n*-9	27.2 ± 1	27.4 ± 1.5	29.1 ± 2.8	30.5 ± 0.6	30.1 ± 1.0	25 ± 4.0	26.4 ± 1	18.4 ± 9.4	29 ± 6.0	24.9 ± 9.1
18:1*n*-7	3.0 ± 0.2	3.0 ± 0.1	2.8 ± 0.2	2.7 ± 0.2	2.9 ± 0.3	2.9 ± 0.1	2.7 ± 1.1	2.6 ± 1.3	3.4 ± 0.1	2.5 ± 1.0
18:2*n-*6	15.5 ± 0.6	15 ± 0.9	15.1 ± 1.9	15 ± 1.3	13.8 ± 1.1	8.9 ± 0.1	10.8 ± 4.1	8.1 ± 5.4	14.5 ± 1.7	11.2 ± 2
18:3*n-*6	0.3 ± 0.0	0.3 ± 0.0	0.3 ± 0.1	0.4 ± 0.0	0.3 ± 0.0	0.1 ± 0.1	0.6 ± 0.6	0.1 ± 0.1	0.4 ± 0.2	0.2 ± 0.1
18:3*n-*3	2.1 ± 0.3	2.0 ± 0.2	2.0 ± 0.2	2.0 ± 0.5	2.0 ± 0.6	0.7 ± 0.1	1.2 ± 1.0	1.3 ± 1.0	2.4 ± 0.6	1.4 ± 1.6
18:4*n*-3	0.8 ± 0.1	0.8 ± 0.1	0.9 ± 0	0.8 ± 0.2	0.7 ± 0.1	0.8 ± 0.0	0.8 ± 0.4	0.7 ± 0.3	0.9 ± 0.3	0.6 ± 0.4
20:0	0.4 ± 0.1	0.4 ± 0.2	1.2 ± 0.6	0.3 ± 0.1	0.3 ± 0.2	0.2 ± 0.1	0.5 ± 0.5	0.1 ± 0.2	0.2 ± 0.3	0.1 ± 0.1
20:1	2.3 ± 0.1	2.3 ± 0.2	2.1 ± 0.2	2.2 ± 0.2	2.4 ± 0.2	1.6 ± 0.1	2.4 ± 2.8	2.0 ± 1.1	2.2 ± 0	2.3 ± 0.4
20:2	0.5 ± 0.1	0.5 ± 0.1	0.5 ± 0.1	0.4 ± 0.1	0.5 ± 0.2	1.4 ± 0.4	0.3 ± 0.3	0.5 ± 0.1	0.4 ± 0.1	0.3 ± 0.2
20:3*n*-6 (DGLA)	0.5 ± 0.2*c*	0.5 ± 0.1*c*	1.4 ± 0.3*b*	2.8 ± 0.4*a*	0.5 ± 0.3*c*	0.5 ± 0.1	0.6 ± 0.3	1.3 ± 1.3	2.6 ± 0.4	1.1 ± 0.7
20:4*n*-6 (ARA)	2.1 ± 0.4*c*	3.3 ± 0.2*a*	0.7 ± 0.5*b*	0.6 ± 0.2*b*	0.4 ± 0.2*b*	1 ± 0.4	2.1 ± 0.8	2.2 ± 2.1	1.8 ± 0.4	1.1 ± 0.8
20:4*n*-3	0.5 ± 0.1	0.5 ± 0.1	0.5 ± 0.1	0.5 ± 0.0	0.5 ± 0.1	0.2 ± 0.0	0.2 ± 0.2	0.1 ± 0.2	0.4 ± 0.7	0.4 ± 0.3
20:5*n*-3 (EPA)	2.6 ± 0.3	2.4 ± 0.4	2.5 ± 0.1	2.5 ± 0.3	2.5 ± 0.5	0.7 ± 0.1	2 ± 0.3	1.2 ± 1.2	2.4 ± 0.4	2.1 ± 0.9
22:0	0.3 ± 0.1	0.3 ± 0.1	0.6 ± 0.4	0.2 ± 0	0.3 ± 0.1	0.6 ± 1.0	0.7 ± 1.0	0.1 ± 0.2	0.0 ± 0.0	0.1 ± 0.1
22:4*n*-6	0.2 ± 0.1	0.2 ± 0	0.1 ± 0.1	0.1 ± 0.1	0.1 ± 0.1	1.3 ± 2.2	0.7 ± 0.7	0.6 ± 1.0	0.2 ± 0.7	0.3 ± 0.2
22:5*n*-6	0.2 ± 0.1	0.2 ± 0.2	0.4 ± 0.1	0.2 ± 0.1	0.2 ± 0.2	0.4 ± 0.4	0.1 ± 0	0.5 ± 0.2	0.3 ± 0.5	0.2 ± 0.2
22:5*n-*3	0.8 ± 0.1	0.7 ± 0	0.7 ± 0.1	0.6 ± 0.1	0.7 ± 0.1	1.3 ± 1.9	0.5 ± 0.4	0.9 ± 0.7	0.6 ± 1.5	0.8 ± 0.3
22:6*n-*3	4.2 ± 0.6	4.1 ± 0.1	4.2 ± 0.4	4.0 ± 0.3	4.3 ± 0.6	1.9 ± 0.5	3.7 ± 0.9	2.4 ± 2.1	3.4 ± 0	3.6 ± 1.8
**FA content (**μ**g mg**^−1^ DW)^*^										
DGLA	0.3 ± 0.1b	0.4 ± 0.2b	0.9 ± 0.4ab	1.8 ± 1.2a	0.4 ± 0.2b	0.3 ± 0.2b	0.2 ± 0.1b	0.3 ± 0.2b	1.4 ± 1.0a	0.2 ± 0.1b
ARA	1.6 ± 0.3ab	2.6 ± 1.8a	0.5 ± 0.6b	0.4 ± 0.2b	0.3 ± 0.2b	0.5 ± 0.3b	0.9 ± 0.2b	0.7 ± 0.2a	0.9 ± 0.1ab	0.3 ± 0.2b
TFA	77.4 ± 26.7	78.4 ± 52.4	66.4 ± 32.1	67.1 ± 51.7	91.3 ± 68.4	43.8 ± 19.6	43.7 ± 12.2	26.4 ± 22.1	50.2 ± 15.7	28.3 ± 22.5

*Data represents fatty acid composition from the neutral lipid fraction before and after S. iniae challenge. Values shown as mean ± SD, n = 20. Different letters a, b and c denote significant (p < 0.05) difference in treatments, as determined by ANOVA*.

* *FA content represents μg of the FA per mg of liver dry weight (DW)*.

**Table 5:** Fatty acid composition (% of TFA) and content in liver polar lipids of zebrafish fed with diets supplemented (w/w) with different levels (7.5 and 10%) of the microalga *Lobosphaera incisa* WT and mutant P127.

**Fatty acid**	**Before challenge**					**After challenge**				
	**WT 7.5 %**	**WT 15 %**	**MUT 7.5%**	**MUT 15%**	**Control**	**WT 7.5 %**	**WT 15 %**	**MUT 7.5%**	**MUT 15%**	**Control**
14:0	1.0 ± 0.3	0.7 ± 0.1	0.8 ± 0.2	0.9 ± 0.3	0.9 ± 0.2	0.7 ± 0.2	0.4 ± 0	1.7 ± 0.3	1.6 ± 0.2	1.5 ± 0.5
16:0	21.3 ± 0.9	21.5 ± 0.4	21.9 ± 0.3	21.7 ± 2.2	21.8 ± 0.8	22.0 ± 1.1	21.2 ± 0.3	25.0 ± 7.1	26.1 ± 11.9	24.7 ± 3.0
16:1*n*-5	0.9 ± 0.7	0.4 ± 0	0.6 ± 0.3	0.4 ± 0	0.6 ± 0.1	0.7 ± 0.1	0.5 ± 0	0.2 ± 0.3	0.4 ± 0	0.8 ± 0.2
16:1*n*-7	1.4 ± 0.5	1.3 ± 0.5	1.2 ± 0.3	1.4 ± 0.4	1.8 ± 0.2	1.8 ± 0.1	1.4 ± 0.1	3.3 ± 2.1	2 ± 0.7	3.2 ± 0.9
16:2	0.4 ± 0.4	0.3 ± 0.4	0.4 ± 0.4	0.4 ± 0.5	0.3 ± 0.4	0.5 ± 0	0.2 ± 0.3	0.5 ± 0.5	0.6 ± 0.3	0.6 ± 0.1
16:2	0.6 ± 0.5	0.6 ± 0.3	0.6 ± 0.3	0.7 ± 0.6	0.6 ± 0.4	0.8 ± 0.3	0.4 ± 0.1	2.3 ± 2.7	1 ± 0.9	0.4 ± 0.2
16:3	0.4 ± 0.1	0.4 ± 0.2	0.4 ± 0	0.4 ± 0.1	0.5 ± 0.1	0.4 ± 0.2	0.3 ± 0.2	2.4 ± 3.2	1.1 ± 0.7	0.6 ± 0.2
16:4	0.2 ± 0.2	0.2 ± 0.2	0.3 ± 0.2	0.4 ± 0.2	0.3 ± 0.1	0.3 ± 0.1	0.1 ± 0	0.9 ± 1.4	0.4 ± 0.4	0.2 ± 0.1
18:0	12.9 ± 2.7	12.0 ± 1.1	12.1 ± 1.2	11.3 ± 1.8	11.0 ± 0.6	11.5 ± 1.2	14.1 ± 0.2	16.5 ± 3.3	10.2 ± 5.7	9.3 ± 1.1
18:1*n*-9	14.2 ± 2.9	14.0 ± 1.4	15.2 ± 1.1	16.0 ± 2.6	17.0 ± 1.3	17.8 ± 1.0	16.1 ± 2.2	17.9 ± 2.9	17.8 ± 7	23.8 ± 4.3
18:1*n*-7	2.0 ± 0.4	2.1 ± 0.3	1.8 ± 0.2	1.9 ± 0.3	2.0 ± 0.2	1.9 ± 0.2	1.7 ± 0.2	2.1 ± 0.4	1.8 ± 0	2.9 ± 1.5
18:2*n-*6	7.5 ± 1.8	7.0 ± 1.1	7.4 ± 1.0	8.3 ± 1.7	8.2 ± 0.7	6.8 ± 0.9	5.8 ± 0.5	8.4 ± 5.1	7.5 ± 4.5	8.7 ± 5.4
18:3*n-*6	0.2 ± 0.1	0.2 ± 0	0.3 ± 0	0.3 ± 0.1	0.2 ± 0.1	0.3 ± 0.1	0.2 ± 0.1	0.1 ± 0.2	0.2 ± 0.2	0.2 ± 0.2
18:3*n-*3	0.9 ± 0.4	0.7 ± 0.1	0.8 ± 0.2	0.9 ± 0.2	0.9 ± 0.2	0.7 ± 0.3	0.2 ± 0.1	1.3 ± 1.5	2.7 ± 2.9	0.8 ± 0.6
18:4*n*-3	0.5 ± 0.2	0.4 ± 0.1	0.5 ± 0.1	0.5 ± 0.2	0.5 ± 0.1	0.5 ± 0	0.3 ± 0.1	1.3 ± 1.4	0.6 ± 0.2	0.6 ± 0.3
20:0	0.5 ± 0.5	0.3 ± 0.1	0.5 ± 0.2	0.4 ± 0.1	0.4 ± 0.2	0.1 ± 0.1	0.1 ± 0	0.1 ± 0.2	0.2 ± 0.1	0.6 ± 0.9
20:1	1.4 ± 0.1	1.3 ± 0.2	1.4 ± 0.2	1.2 ± 0	1.3 ± 0.3	1.2 ± 0.1	1.2 ± 0.1	1.0 ± 0.7	3.4 ± 1.7	1.4 ± 0.9
20:2	0.7 ± 0.2	0.6 ± 0.1	0.5 ± 0.1	0.5 ± 0.1	0.6 ± 0.1	0.6 ± 0.1	0.6 ± 0	1.5 ± 1.8	0.9 ± 1.0	0.4 ± 0.1
20:3*n*-6 (DGLA)	0.6 ± 0.1b	0.5 ± 0.1b	2.6 ± 0.4*c*	4.0 ± 0.5a	0.9 ± 0.3b	1.2 ± 0.2	0.9 ± 0	2.6 ± 1.1	2.2 ± 0.4	0.7 ± 0.3
20:4*n*-6 (ARA)	8.8 ± 1.1c	11.3 ± 0.7a	3.4 ± 0.1b	3.4 ± 0.6b	2.2 ± 0.4b	6.9 ± 1.8	11.3 ± 0.7	1.2 ± 0.8	2.8 ± 2.6	1.4 ± 1.0
20:3*n*-3	0.3 ± 0.1	0.2 ± 0.1	0.3 ± 0.2	0.2 ± 0.1	0.2 ± 0	0.3 ± 0.3	0.1 ± 0.1	0.5 ± 0.7	0.5 ± 0.5	0.2 ± 0.1
20:4*n*-3	0.5 ± 0.1	0.3 ± 0.1	0.6 ± 0.6	0.4 ± 0	0.4 ± 0	0.2 ± 0.1	0.2 ± 0	0.5 ± 0.6	0.2 ± 0.2	0.3 ± 0.1
20:5*n*-3 (EPA)	2.9 ± 0.5b	2.4 ± 0.3b	3.9 ± 0.6a	3.5 ± 0.4a	4.1 ± 0.8a	2.8 ± 0.5	2.0 ± 0.4	1.0 ± 0.5	1.9 ± 1.5	2.5 ± 1.2
22:0	0.4 ± 0.3	0.3 ± 0.1	0.4 ± 0.1	0.3 ± 0.1	0.2 ± 0.1	0.4 ± 0.2	0.1 ± 0.1	0.3 ± 0.3	1.6 ± 2.1	0.8 ± 1.1
22:4*n*-6	0.6 ± 0.1ab	1.0 ± 0.3a	0.4 ± 0.1b	0.3 ± 0.1b	0.4 ± 0.1b	0.6 ± 0.2	0.8 ± 0	0.1 ± 0.1	2.5 ± 0.1	0.4 ± 0.2
22:5*n*-6	0.5 ± 0.2	0.5 ± 0.2	0.6 ± 0.3	0.5 ± 0.3	0.5 ± 0.3	0.4 ± 0	0.5 ± 0.1	0 ± 0	0.9 ± 1.0	0.3 ± 0.2
22:5*n*3	1.2 ± 0.2	1.1 ± 0.2	1.2 ± 0.1	1.2 ± 0.3	1.4 ± 0.2	1.5 ± 0.3	1.3 ± 0.3	1.9 ± 1.8	0.6 ± 0.6	1.1 ± 0.4
22:6*n*3	16.3 ± 1.7	17 ± 1.9	19.2 ± 2.0	17.0 ± 3.0	19.6 ± 3.3	16.8 ± 0.7	17.4 ± 1.6	3.9 ± 4.2	5.2 ± 5.5	8.9 ± 6.4
**FA content (**μ**g mg**^−1^ DW)^*^										
DGLA	0.2 ± 0.1b	0.3 ± 0.2b	0.7 ± 0.2b	1.7 ± 0.9ab	0.4 ± 0.1a	0.2 ± 0b	0.1 ± 0b	0.6 ± 0.3a	0.4 ± 0ab	0.1 ± 0b
ARA	2.8 ± 0.8ab	5.3 ± 3.0a	1.0 ± 0.4b	1.5 ± 0.9b	0.9 ± 0.2b	1.0 ± 0.2a	1.6 ± 0.4a	0.2 ± 0.2b	0.3 ± 0b	0.2 ± 0.1b
TFA	32.9 ± 9.2	47.2 ± 26.9	28.9 ± 11	42.6 ± 21.4	39.2 ± 6.1	15.4 ± 2.4	14.1 ± 2.6	22 ± 1.6	15.3 ± 0	16.1 ± 9.2

*Data represents fatty acid composition from the polar fraction before and after S. iniae challenge. Values shown as mean ± SD, n = 20. Different letters a, b and c denote significant (p < 0.05) difference in treatments, as determined by ANOVA*.

* *FA content represents μg of the FA per mg of liver dry weight (DW)*.

### Effects of dietary treatment and resistance to *Streptococcus iniae* challenge

**Figure 2:**
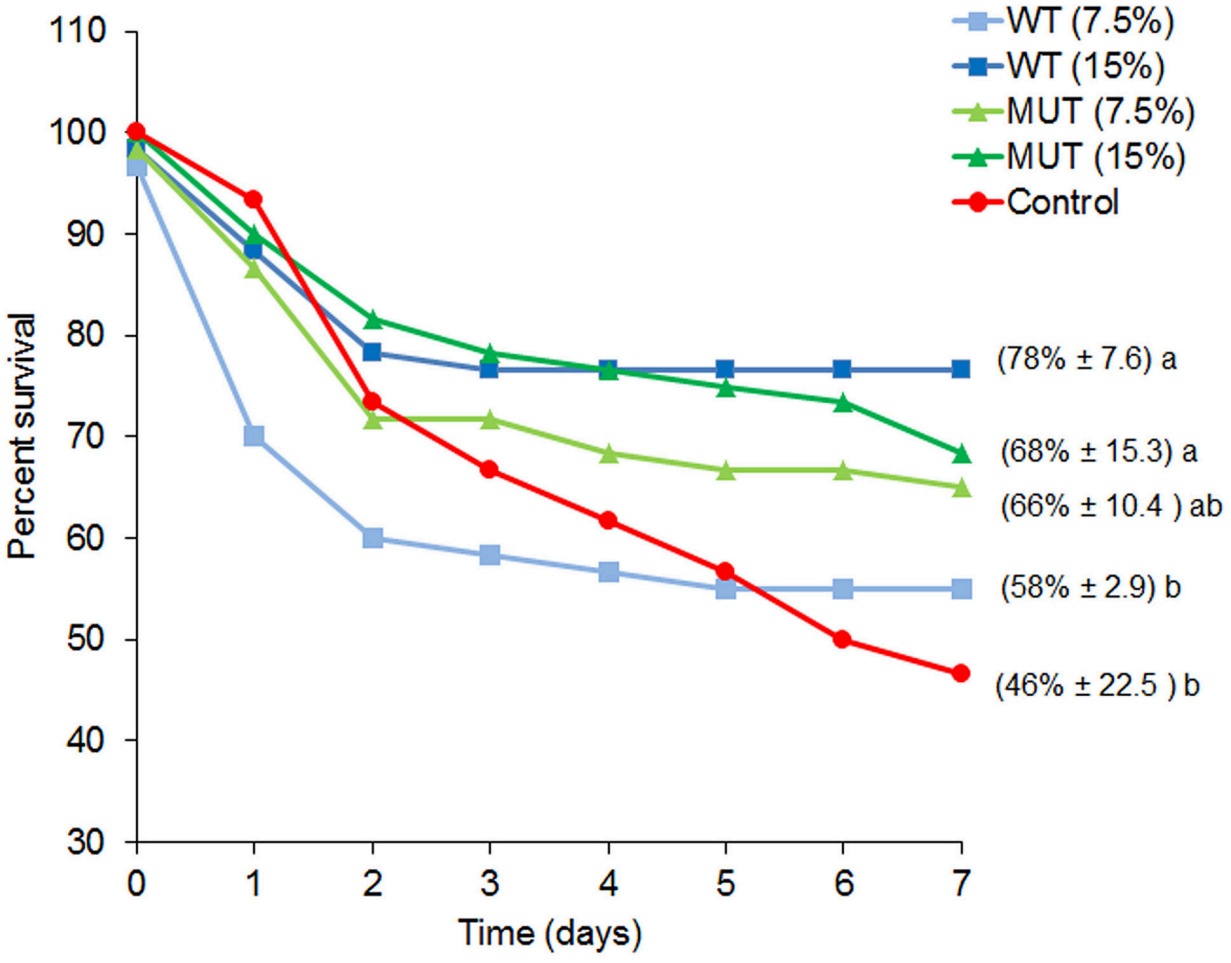
Percent survival for each experimental dietary treatment following *S. iniae* challenge. Data represent the mean percentage of survival during the course of the challenge (*n* = 4, 20 fish/tank). Numbers in brackets indicate survival percentage ± SD, and different letters (a, b) indicate significant (ANOVA, *p* < 0.05) differences in survival between treatments.

To examine the effect of dietary manipulations on disease resistance, fish were challenged with *S. iniae* after completing 1 month of dietary supplementation, and mortality was recorded for a period of 1 week (Figure 2 and Supplementary Figure 1). Overall, fish fed with supplemented diets showed elevated survival as compared to fish fed with control feed. Survival of fish fed with 15% of both WT- and MUT-supplemented diets was significantly (*p* < 0.05) higher as compared to the control (78 and 68% in WT and MUT, respectively, as compared to 46% in the control).

### Expression of immune-related genes in the kidney

**Figure 3:**
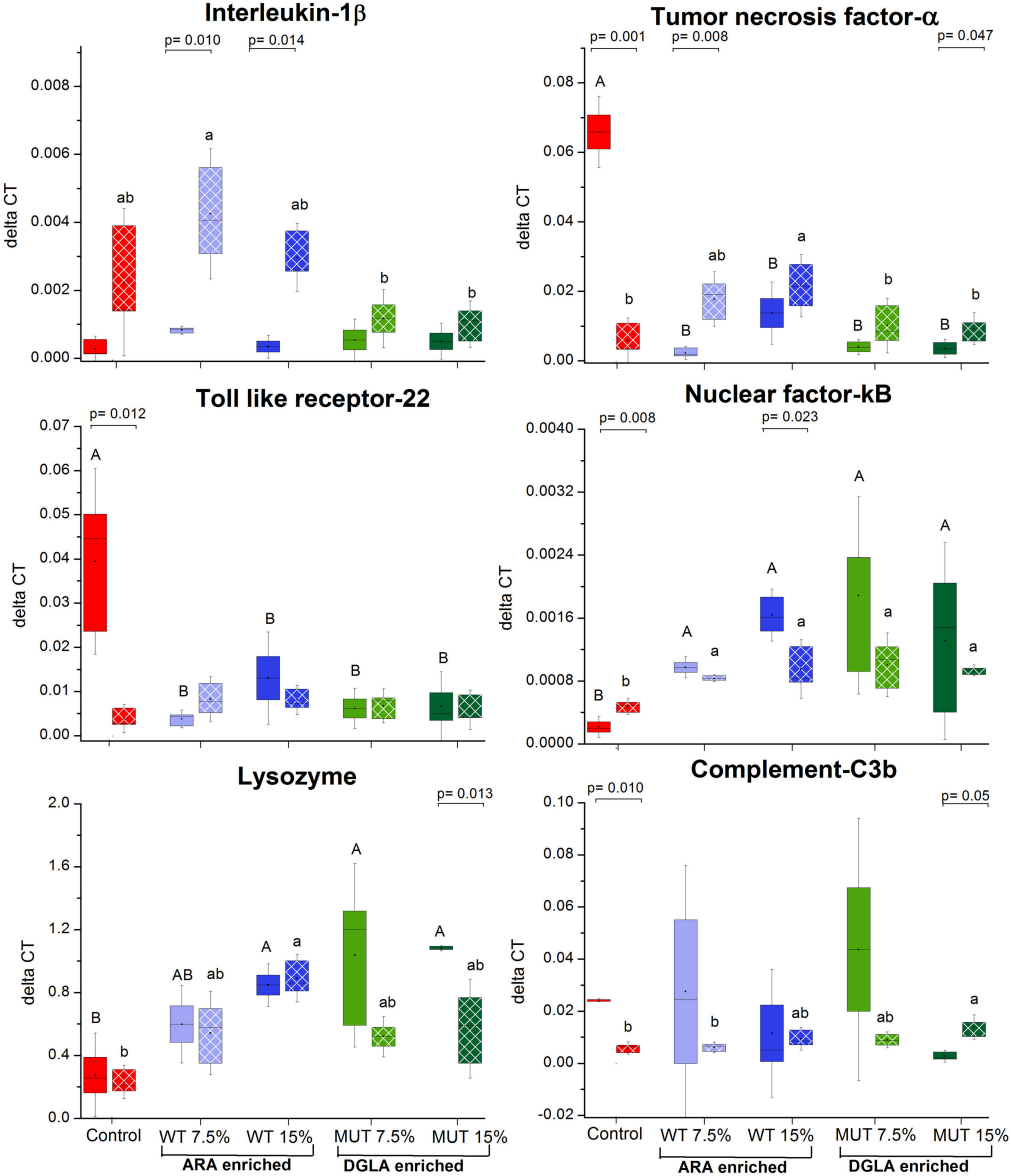
Real-time PCR quantification of expression of genes involved in immune responses in zebrafish kidneys. Data are presented as ΔCT (delta cycle threshold, expression normalized to housekeeping gene EF1) using box and whisker plots. Expression of genes before and after challenge is represented by solid colored and pattern-filled boxes, respectively (*n* = 4 biological replicates, a pool of five fish each). Significant difference between treatments before and after challenge is indicated in bracket with corresponding p value, as determined by *t*-test. A and B denote significant differences between treatments before challenge; a and b denote significant differences between treatments after challenge. Differences were considered significant at *p* < 0.05.

**Figure 4:**
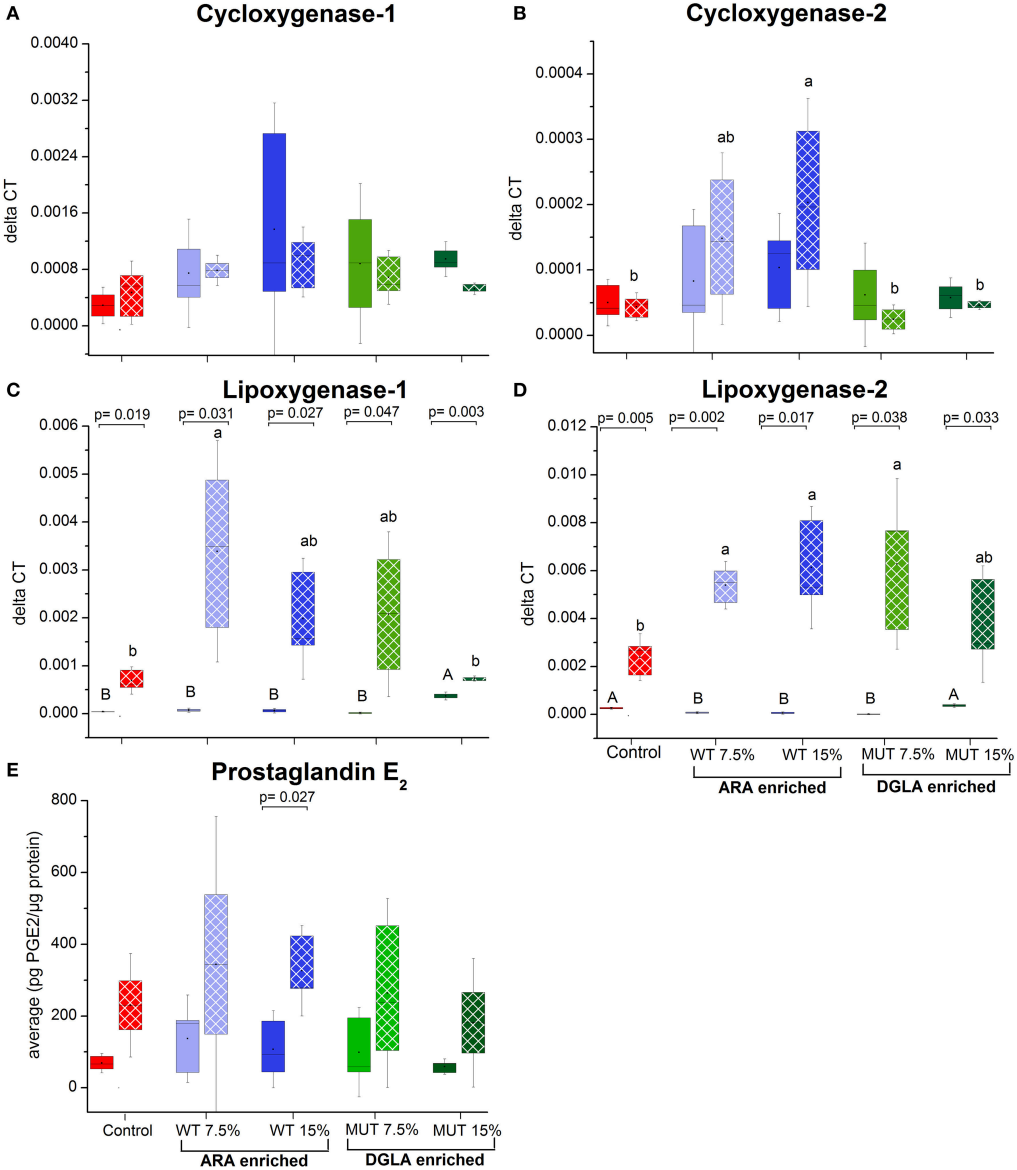
Expression of genes associated with eicosanoid synthesis and prostaglandin E_2_ production in zebrafish kidneys. **(A–D)** Real-time PCR quantification of expression of selected genes involved in eicosanoid synthesis in zebrafish kidneys. Data are presented as ΔCT (delta cycle threshold, expression normalized to housekeeping gene EF1) using box and whisker plots. Expression of genes before and after challenge is represented by solid colored and pattern-filled boxes, respectively (*n* = 4 biological replicates, a pool of five fish each). Significant difference between treatments before and after challenge is indicated in asterisk bracket with corresponding p value, as determined by *t*-test. A and B denote significant differences between treatments before challenge; a and b denote significant differences between treatments after challenge. Differences were considered significant at *p* < 0.05. **(E)** Prostaglandin E_2_ levels in kidneys of zebrafish. ELISA was used to quantify PGE_2_ concentration in homogenized kidney samples obtained from zebrafish fed with different diets. Box plot represents average pg PGE_2_/μg protein ± SD (*n* = 15, 3 biological replicates/treatment) for each dietary treatment. PGE_2_ levels before and after challenge represented by solid-colored and pattern-filled boxes, respectively. Significant difference (*p*-values of *t*-test) between treatments before and after challenge is indicated by bracket.

To evaluate the impact of dietary supplementation on the expression of genes related to immune function and eicosanoid production, a quantitative real-time PCR was performed using RNA isolated from the kidneys of zebrafish fed with the different experimental diets for a period of 1 month (Figures 3, 4 and Supplementary Figure 2). Results indicated a significant increase in the gene expression of the nuclear transcription factor gene NFκB, a major master regulator of inflammatory response, in fish fed with both WT- and MUT-supplemented diets, as compared to the control (Figure 3). However, the expressions of other examined genes, including *TLR 2*2, a membrane receptor activated by pathogen-associated molecular patterns (PAMPs) and the pro-inflammatory cytokine TNF-α, were significantly downregulated in fish fed with both supplemented diets, as compared to the control. The gene expression of another pro-inflammatory cytokine, interleukin 1β, did not change between treatment groups (Figure 3). The microalgal supplemented diets also upregulated the gene expression of lysozyme, an important innate immune factor in fish, but did not affect expression of the complement C3B gene, another key factor in fish's innate immune system (Figure 4). The analysis of the gene expression of enzymes that convert *n*-6 LC-PUFA to eicosanoids is presented in Figure 4. Although not statistically significant, the expression of *COX-1* and *COX-2* was elevated in the fish groups fed all the experimental diets, regardless of their ARA or DGLA contents, compared to the controls. The expression of *LOX-1* (a homolog of the human *LOX-15* gene) was moderately elevated only in the MUT 15% supplemented group, whereas the gene expression of *LOX-2* (a homolog of the human *LOX-5* gene, involved in ARA conversion to leukotrienes) was downregulated in the WT-supplemented group (Figure 4).

### Expression of genes related to ARA metabolism and antioxidant defense in the liver

**Figure 5:**
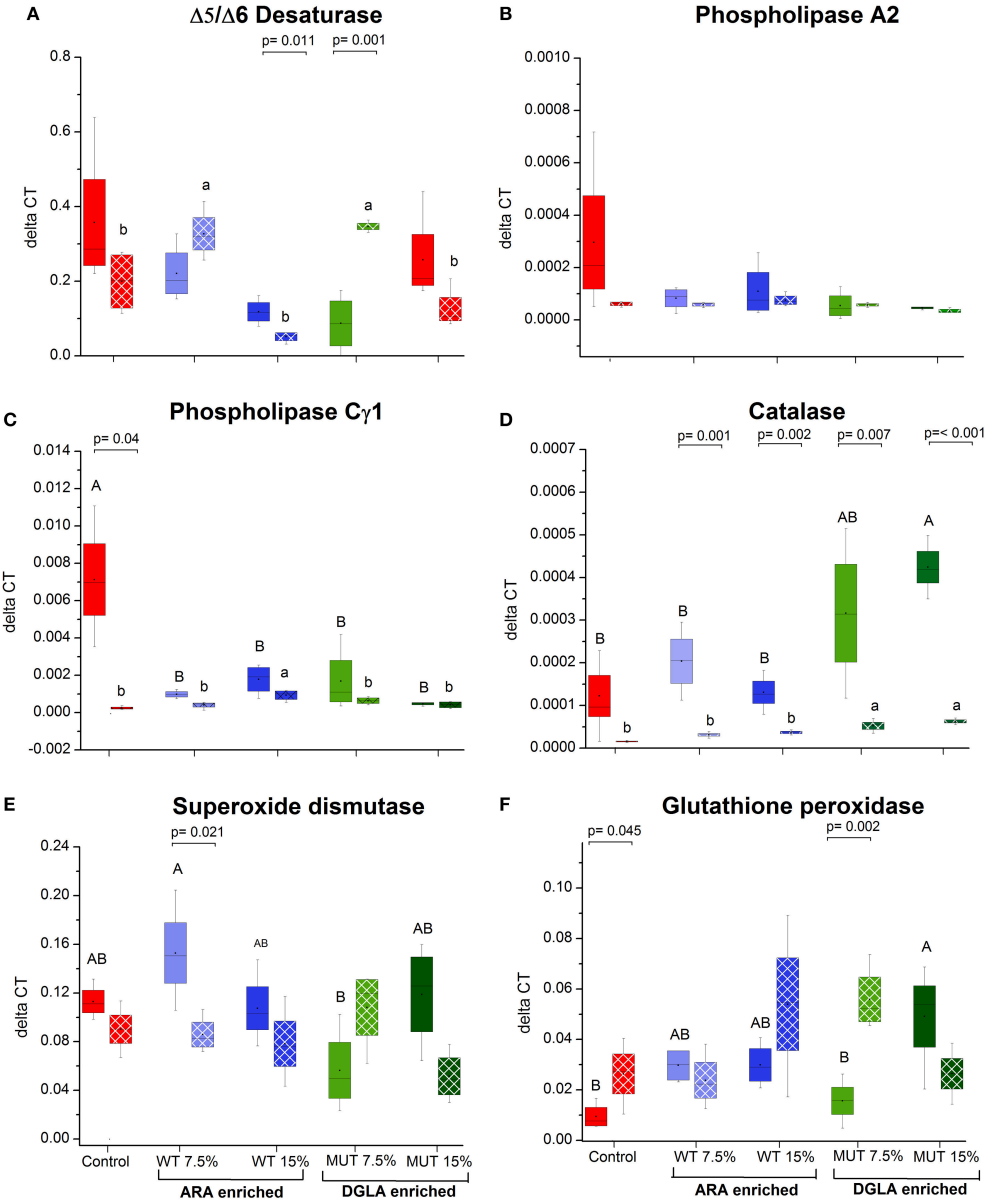
Gene expression in zebrafish liver. **(A–C)** Expression of genes related to ARA metabolism in zebrafish liver, **(D–F)** expression of genes involved in antioxidant defense. Data are presented as ΔCT (delta cycle threshold, expression normalized to housekeeping gene EF1) using box and whisker plots. Expression of genes before and after challenge is represented by solid colored and pattern-filled boxes, respectively (*n* = 4 biological replicates, a pool of five fish each). Significant difference between treatments before and after challenge is indicated in bracket with corresponding p value, as determined by *t*-test. A and B denote significant differences between treatments before challenge; a and b denote significant differences between treatments after challenge. Differences were considered significant at *p* < 0.05.

As a major organ involved in lipid metabolism and LC-PUFA biosynthesis, the liver was analyzed for several selected genes that are involved in ARA biosynthesis and its release from cell membranes. All the analyzed genes, including Δ5/Δ6 desaturase, phospholipase C-γ1 (PLC γ) and phospholipase A2 (PLA_2_), were downregulated in the liver of fish fed with the experimental diets, although differences were significant only for the phospholipase C-γ1 gene (Figures 5A–C, Supplementary Figure 3). Antioxidant defense genes were analyzed in the liver, as a major organ for antioxidant production and ROS scavenging (34) (Figures 5D–F, Supplementary Figure 3). Comparing the expression level to the control, supplemented diets led to a significant increase in the expression of glutathione peroxidase and catalase, in particular, in the 15% MUT-supplemented diet. When examining the fold-change from the control, differences in catalase gene expression were evident in both MUT-supplemented groups, and for glutathione peroxidase expression, also in the WT-supplemented groups (Supplementary Figure 3). There was no effect on the expression of Cu/Zn superoxide dismutase (Figure 5E, Supplementary Figure 3).

### Changes in fatty acid composition post-infection

ARA in neutral lipids decreased in surviving fish fed with WT-supplemented diets, while those fed with MUT-supplemented diets showed an increase (Table 4, Figure 1). Levels of DGLA post-infection did not vary within treatment groups and maintained a trend similar to before challenge. In polar lipids, ARA decreased in all treatment groups, while DGLA did not change in the WT-fed group but decreased in the 15% MUT-fed and control group (Table 5, Figure 1).

### Expression of immune-related and eicosanoid synthesis genes in fish surviving infection

The expression of specific genes in the kidneys of surviving fish was measured after 1 week of *S. iniae* challenge to evaluate the potential role of target gene expression during recovery following microbial challenge and the late stages of infection. Results revealed significant modulation in the expression patterns of the examined genes in recovering fish, suggesting an effect of dietary supplementation on the fish response to infection, in general, and on the transcriptional responses, in particular. The expression of interleukin 1β, which was unaffected by the supplemented diets before challenge, showed significantly higher expression in fish fed with WT-supplemented feed post-infection, as compared to the pre-infection expression level (Figure 3). Elevation in expression was also evident in the control, although the difference was not significant. Interestingly, interleukin 1β expression was unaffected by infection in fish fed with the MUT-supplemented feed, as expression levels were similar before and after *S. iniae* infection. The microbial challenge appeared to affect the expression of TNFα, though changes differed between treatment groups. The *S. inaie* challenge significantly decreased the expression of the TNFα gene in fish fed with the control feed, whereas the expression was elevated in fish fed with microalgae-supplemented diets (significantly for 7.5% WT- and 15% MUT-fed groups). The overall expression of TNFα was significantly higher in fish fed with WT than in fish fed with MUT and the control diets (Figure 3). The *TLR-22* expression pattern was similar to that of *TNF*α. The TLR-22 gene was significantly downregulated in fish fed with the control feed post-infection, while its expression was unaltered in all treatment groups (Figure 3). The expression of NF-κB post-challenge decreased compared to pre-challenge in WT- and MUT-supplemented groups (significantly only in fish fed with 15% WT; Figure 3). The opposite pattern was evident in the control, where a significant increase in expression was recorded. Despite the post-challenge increase in the control and decrease in supplemented groups, the expression level in all supplemented groups remained higher than in the control, as was also seen before the challenge was applied (Figure 3). The expression of the lysozyme-coding gene post-challenge was similar to the pre-challenge level in the control and the WT-fed fish. In the MUT-fed fish, expression decreased post-challenge, with significant differences in the 15%-supplemented fish (Figure 3). The expression of the complement *C3B* gene after challenge was downregulated in the control fish. Expression was generally reduced, though not significantly, in the WT- and 7.5% MUT-fed fish, but was upregulated in the 15% MUT-fed fish (Figure 3). The microbial challenge did not alter the expression of the *COX-1* gene in any of the dietary treatment groups, as expected for the constitutively expressed *COX 1* (Figure 4). In contrast, the elevated expression of the *COX-2* gene was determined in fish fed with WT-supplemented feed, although statistical significance could not be established (Figure 4). In addition, the *LOX-1* and *LOX-2* genes were significantly upregulated post-challenge in all treatment groups including the control (Figure 4). The *LOX1* and *LOX 2* expression levels were noticeably higher in fish fed with supplemented diets than in fish fed with the control diet (Figure 4).

### Expression of ARA metabolism and antioxidant enzymes in fish surviving infection

The expression of three examined genes involved in ARA metabolism in the liver decreased post-challenge in the control group, with significant differences in the PLA2 and PLCγ1 (Figure 5), whereas expression of these genes did not significantly change in the groups fed with supplemented diets (Figure 5). The gene expression of the antioxidant enzyme catalase decreased in all treatment groups following infection, with the significant downregulation of catalase occurring in all groups, except the control (Figure 5). Interestingly, post-infection fish fed with the MUT-supplemented diets had slightly higher expression of catalase than control and WT-fed groups (Figure 5). The expression of glutathione peroxidase and superoxide dismutase did not significantly change post-challenge, apart from a significant decrease in the 7.5% WT-fed fish (Figure 5).

### PGE2 production in kidneys post-infection

Results of measurement of prostaglandin E_2_ production using an enzyme-based immunoassay method are shown in Figure 4E. The PGE_2_ levels were similar between treatment groups before infection. The levels were slightly higher in all groups 9 d post-challenge in surviving fish, with a significant change in the control and 15% WT-fed groups.

### Histopathological analysis

**Figure 6:**
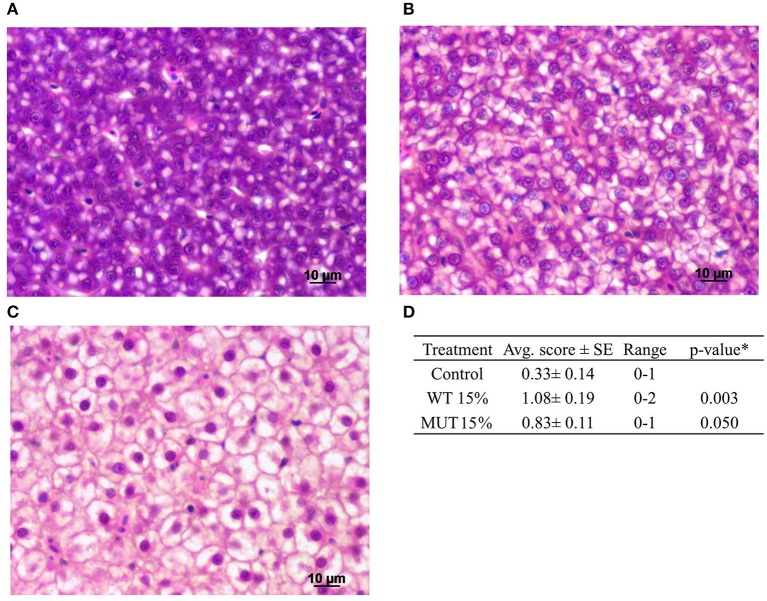
Histopathological analysis of zebrafish liver after feeding with modified experimental diets. Liver cells showing varying degrees of vacuolization are presented: **(A)** normal, scored 0; **(B)** moderate, scored 1; **(C)** high, scored 2. **(D)** Summary of quantitative analysis of the histological findings, showing average (Avg. score) and range of scores for the different treatment groups (*n* = 12 fish per treatment). *To compare treatment groups with the control, a one-way ANOVA was used.

A comprehensive histopathological analysis did not reveal any substantial pathology in any of the organs, except for evident liver vacuolization, which was scored in sampled fish. Images of the different vacuolation levels and the corresponding scores are shown in Figures 6A–C along with a summary of the quantitative analysis (Figure 6D). Based on the liver scoring, fish fed with 15% WT- and 15% MUT-supplemented feeds showed significantly higher degrees of vacuolization with average scores of 1.08 and 0.83, respectively, as compared to a score of 0.33 in the control.

### Multivariate analysis (PCA) of diet impact on gene expression

**Figure 7:**
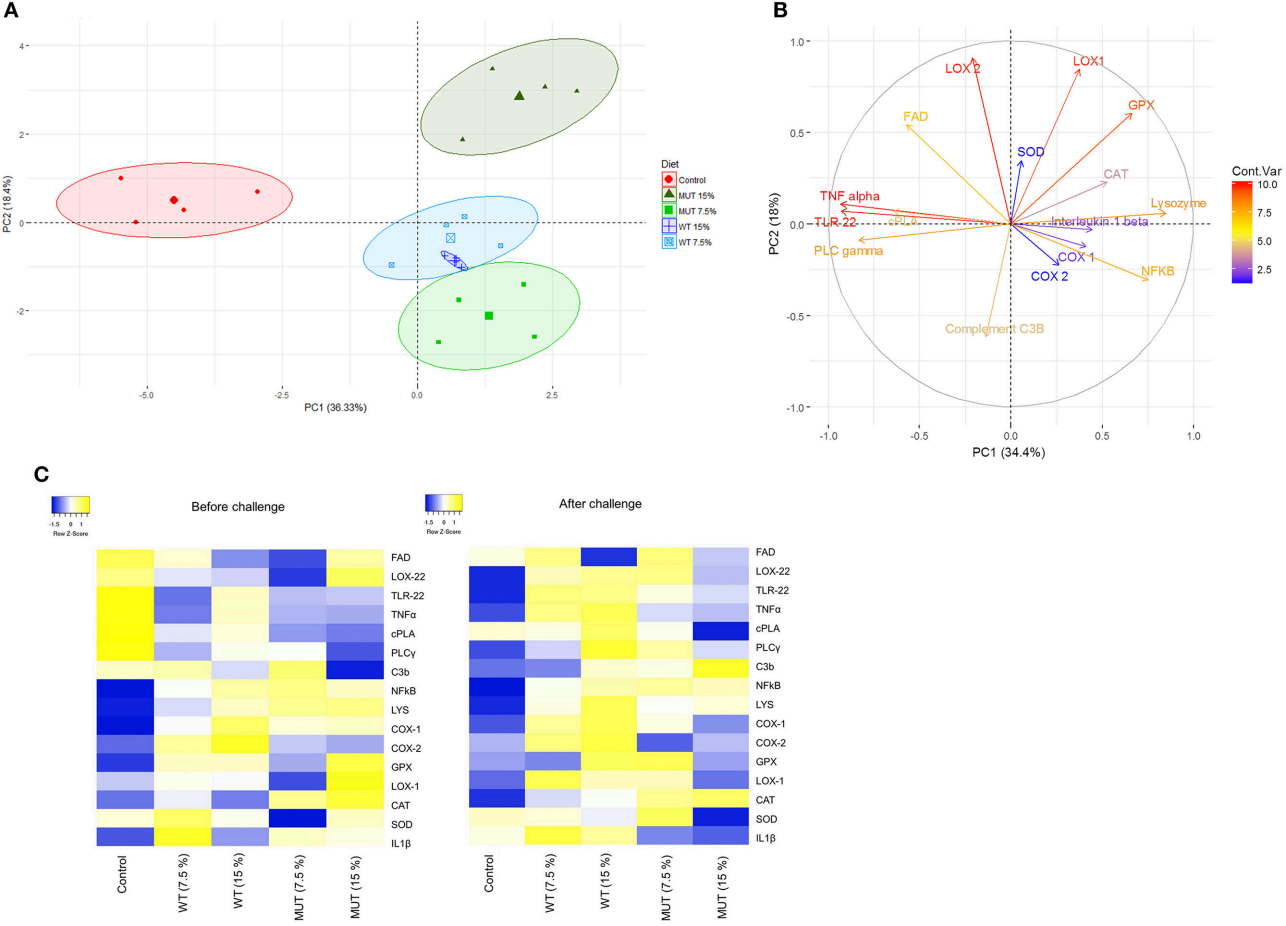
Principle component analysis and heat map of gene expression analysis. **(A)** PCA of individuals. Colored points inside the clusters represent individual biological replicates from different dietary treatments. Values indicated on the axis of the factor map correspond to the percentage of total variance explained by each axis (PC1 and PC2, respectively). **(B)** PCA of variables. Colored lines with gene labels and intensity scale represent the contribution of each variable (gene) to the treatment. **(C)** Heat map showing expression of genes analyzed in the liver and kidneys of zebrafish fed with different diets. Plots represent average of log 2-fold expression ratios (normalized to internal control Elf2) of genes in different treatments (four biological replicates per treatment). Levels of downregulation (blue) and upregulation (yellow) are shown on a log 2 scale.

A principal component analysis (PCA) was performed on the normalized expression values obtained from the qPCR, to visualize patterns of gene expression and its modulation under different dietary treatments. Data are displayed in a scatter plot view with different colors representing the dietary treatment (Figure 7A). The biological replicates for each treatment occupied similar places with close clustering, indicating consistency between replicates. Results from the individual PCA plot revealed clear separation between the control group and the supplemented groups (Figure 7A). The first two components, PC1 and PC2, explained 54% (36.3 and 18.4%, respectively) of the variance, while PC3 explained 10.20% (Figure 7A and Supplementary Figure 4). Overall, three components from this PCA explained more than 60% of the variance in the data. The contribution of variables (gene expression) to the dietary treatments is shown with the help of the variable factor map, where the degree of each gene's contribution is represented by the length of the vector and intensity scale marked on the legend (Figure 7B). Genes encoding LOX2, LOX1, TNF, TLR22, NF-kB, lysozyme, GPX, and PLCγ displayed higher contributions, indicating their significant modulation in expression because of the dietary treatments. At the same time, genes such as *COX1, COX2, IL1*β, and *SOD* were the least affected by the dietary treatments. The biplot clearly shows that genes encoding lipoxygenases (*LOX1* and *LOX2*) and *GPX* were affected by the 15% MUT- supplemented diets, whereas genes such as *TNF*α*, TL22, PLA2*, and *PLC*γ were affected by the control diet.

## Discussion

Studies have established the role of ARA as a pro-inflammatory LC-PUFA, while DGLA has been associated with relieving inflammation (6, 19). However, in various pathopyhisiological conditions, the distinction between these *n*-6 LC-PUFA's pro- and anti-inflamatory effects may not be that apparent. Furthermore, ARA metabolism also generates resolving molecules, such as lipoxin A4, essential for inflammation resolution. The *n*-6 LC-PUFA-associated metabolic pathways that lead to the distinct immunomodulatory effects involve eicosanoid signaling, which modulates a range of physiological conditions related to immune response and inflammation. Omega-6 LC-PUFA and their derivatives are capable of modifying cell membrane composition and the regulation of target transcription factors and host gene expression (35, 36). In this study, changes in fatty acid composition and gene expression modulation were revealed in zebrafish following dietary supplementation with the microalgae *L. incisa*, rich in ARA or DGLA. Importantly, microalgae-supplemented diets demonstrated a positive effect on resistance to bacterial challenge, suggesting that such a nutrutional manipulation may be an efficient approach to improving the immune status and resistance to bacterial infection in fish.

In this work, zebrafish were fed with experimental diets, in which a certain fraction of the original feed (7.5 and 15%) was replaced with broken biomasses of two strains of the oleagenous microalga *L. incisa*. The two strains of this microalga accumulate *n*-6 LC-PUFA as the major component of triacylglycerols, in particular under nitrogen starvation (23, 37). Hence, *n*-6 LC-PUFA-enriched triacylglycerols were the major source of dietary ARA and DGLA, in the WT- and MUT-supplemented diets, respectively. Based on the alterations in the fatty acid profile of liver neutral and membrane lipids, and their enrichment with the respective dietary microalgae-derived *n*-6 LC-PUFA, our results indicate that zebrafish fed on the microalgae-supplemented diets efficiently incorporated the fatty acids provided via broken biomass supplementation. The enrichment was most apparent in the polar lipid fraction that represents the membrane-associated lipids, implying that the dietary manipulations influenced the composition and properties of the cellular membranes and the availability of *n*-6 LC-PUFA in both groups, ARA and DGLA, for the formation of lipid mediators such as eicosanoids. Similar changes in the fatty acid profile were previously reported in the liver of guppies fed the ARA-rich *L. incisa* WT as a dietary supplement (11). It is important to note that the enrichment with microalgae-derived *n*-6 LC-PUFA affected the proportion of *n*-3 EPA in the polar lipid fraction, but not of DHA, indicating that DHA biosynthesis was not affected. Enrichment with the microalgae was associated with elevated liver vacuolation, showing lipid deposition in hepatocytes. It should be noted that the vacuolation level, even at the highest grade, was not high enough to raise pathological concerns, and is not expected to be associated with adverse health-related consequences. The elevated lipid deposition can be attributed to the higher lipid content in experimental diets (Table 3).

As a major hematopoietic organ, the kidneys contain large numbers of mature, active leucocytes (38) and were therefore selected for analysis of immune-related genes. Our findings showed substantial modulation in the expression of immune and inflammation-related genes in the kidneys of zebrafish fed with WT- and MUT-suplemented diets, compared to fish fed with unsupplemented feed (Figures 3, 4). This general effect was clearly evident in the generated heatmap (Figure 7C).

Generally, *n-*6-PUFA and their metabolites are well known to affect gene expression by activating nuclear factors such as NF-κB (36). NF-κB in its native state exists as a heterodimer consisting of the subunits P50 and P65 bound to the inhibitor IκB. The phosphorylation and proteolytic degradation of IκB results in its activation and translocation to the nucleus, where it regulates gene transcription (39). ARA, regarded as a pro-inflammatory LC-PUFA, is therefore expected to induce upregulation of this gene; however, its elevated expression in the DGLA-rich diet-fed fish is intriguing, considering its anti-inflammatory properties. These findings may indicate that DGLA's anti-inflammatory action is uncoupled from the NF-κB upregulation. ARA was shown to induce NF-κB translocation and activation of human promonocytic cell lines, as well as macrophages (40) and in enterocytes (41). However, in both these studies, the activation was mediated through the production of the prostaglandin PGE_2_. It seems likely that DGLA or its derivatives (e.g., PGE_1_) exert an effect on NF-κB expression similar to ARA. In the present study, there was no evident increase in PGE_2_ following dietary supplementation with any of the experimental diets. The lack of increase in PGE_2_ is in line with the lack of changes in COX 1 and 2 gene expression, which are enzymes that produce prostaglandins from ARA and DGLA. In sharp contrast to the upregulation of NF-κB, all the other inflammation-related genes, the cytokines TNFα and IL-1ß and the membrane-receptor TLR-22, were either downregulated or unaffected by the supplemented diets. This clearly suggests that the expression of NF-κB was not as a result of the canonical TNF activation pathways, as cytokines were suppressed with the supplemented diets. This important finding may indicate the beneficial effect of microalgae-supplemented feeding following the bacterial challenge. Although the exact mechanisms for activation of NF-κB by ARA- and DGLA-rich microalgae remains unclear, an important factor that may induce its expression could be the increase in ROS and oxidative stress due to highly PUFA-rich diets (16) or the generation of non-enzymatic lipid peroxidation products. Similar results of decreased expresion of *TNF*α and *IL-*6 following dietary supplementation with elevated ARA were reported in grass carp kidneys (15), although unlike our study, Tian et al. reported the elevated expression of TLR22 in the kidneys of supplemented fish. A similar effect of an almost 60% reduction in TNFα was reported in human blood mononuclear cells, following supplementation with oil rich in γ-linolenic acid (42). Dietary supplementation with WT and MUT resulted in the increased expression of lysozyme in the kidneys, linked to the elevation in NFκB expression, as stated above. Lysozyme is one of the key innate immune factors in fish and is actively secreted from various sites, including lymphoid tissue, macrophages, mucus and plasma (43, 44). An increase in lysozyme activity, along with enhanced survival, was recorded in our earlier study with guppy fry that were fed with artemia enriched in WT *L. incisa* (14). Furthermore, enhanced lysozyme activity with ARA-suplemented diets is documented in recent studies on rabbitfish, Atlantic salmon and grass carp (15, 31, 45). Lysozyme activity is an important index of innate imunity in fish and is well recognized as a useful indicator of fish health (46). Although WT- and MUT-supplemented feeds resulted in increased lysozyme expression, their effects were not evident in the expresion of the complement C3b gene, which remained similar among treatment groups.

In this study, supplemented diets altered the expression of genes encoding enzymes involved in arachidonic acid metabolism. No significant change in the expression of the cyclooxygenase genes (*COX 1* and *COX 2*) was observed. These are in line with the unchanged PGE_2_ levels prior to infection, as determined by EIA. However, an increase in *LOX1* expression, which is homologous to 15-LOX in humans, was noticed in the kidneys of 15% MUT-fed fish. This could be indicative of the enhanced conversion of DGLA to 15-(S)-hydroxy-8, 11, 13-eicosatrienic acid (15-HETrE), which is catalyzed by 15-LOX. The 15-HETrE effects are reported to be associated with suppression of chronic inflammation, vasodilation, lowering of blood pressure, arresting of cancer cell growth and differentiation of tumor cells (19). The enhanced expression of 15-LOX could contribute to imparting the higher anti-inflammatory potential in fish fed with MUT-supplemented diets, which exhibited significantly less mortality in the challenge trials. Dietary *n*6-PUFA and *n*3-PUFA are known to regulate the expression of Δ5-desaturase, which catalyzes the essential step in ARA biosynthesis from DGLA (47, 48). The decrease in this gene's expression in the WT-supplemented fish is likely due to the exogenous supply of ARA. In addition, a substantial decrease in cytosolic Ca^+2^-dependent phospholipase A2 (cPLA2) was evident in the WT- and MUT-supplemented groups. Phospholipase A2 is mostly responsible for releasing ARA from membrane phospholipids. Similarly, (49) reported that PLA2 expression decreased in *Senegalese sole* fed with ARA-supplemented diets and subjected to short-term acute stress. In a study with gilthead seabream larvae, downregulation of cPLA2 was reported as an adaptation to higher ARA levels (13). Similar to cPLA2, a significant decrease in PLC_γ_ was also observed in fish fed with WT- and MUT-supplemented feed. PLC_γ_ is an important member of the lipid metabolism pathways, which hydrolyzes phospholipids to release the potent intracellular signaling messengers diacylglycerol and inositol triphosphate (IP_3_). The release of DAG and IP is responsible in the downstream events that lead to the activation of transcription factors and cytokines (50, 51). Different inositol phosphates are known to directly affect intracellular signaling events (50). A similar inhibition of PLCγ with dietary fish oil rich in *n-*3-LC-PUFAs has been reported in lymphocytes (52, 53); however, in this study, we observed this effect with *n-*6-LC-PUFA-rich feeds. A higher expression of PLCγ has been reported in cancer cells where it acts as a signaling intermediate for cytokines such as interleukins, and it was also reported to promote tumorgenesis through intracellular and extracellular signaling pathways (54, 55). Overall, the suppression of both phospholipases cPLA2 and PLCγ observed in this study is an important indicator of the anti-inflammatory effects of the *n-*6 LC-PUFA ARA and DGLA, and correlates with the reduced expression of pro-inflammatory TLR22 and TNFα.

The 15% MUT-supplemented diet resulted in elevated gene expression of catalase and glutathione peroxidase pre-challenge. Both catalase and glutathione peroxidase remove the H_2_O_2_ generated from the dismutation of the superoxide radical (${\text{O}}_{2}^{-}$), whereas superoxide dismutase enzymes are responsible for maintaining the steady state concentration of ${\text{O}}_{2}^{-}$ (56). Expression of these antioxidant enzymes, which quench free radicals, are essential for immune function, defense against infectious organisms and the regulation of signaling processes (57). Interestingly, the expression of superoxide dismutase, a prime antioxidant enzyme responsible for defense against free radicals, was not affected by our dietary treatments. The elevated expression of the catalase and glutathione peroxidase enzymes may have contributed to the improved fish survival following the *S. iniae* challenge.

The mortality results following the *S. iniae* challenge suggest that the immune modulation induced by experimental diets effectively protected the fish from infection, although it is evident that the pre-challenge immune modulation induced by the treatments differed between the WT- and MUT-supplemented groups. The gene expression analysis occurring 1 week after challenge, when mortalities subsided, represents the period of recovery from infection. The elevated lysozyme expression, at the recovery from infection stage in the WT-supplemented fish, was in line with the elevated expression of the *COX-2* gene, which is generally associated with a pro-inflammatory effect. Changes in gene expression of TNF-α, TLR-22, and NF-κB in the control were in line with the pre-challenge levels in the treatment groups, which were better protected from the infection. It is difficult to point which of the genes could directly explain the higher survival in the treatment groups. It is likely that a combined effect of the various immune factors produced the final protective outcome.

Our results showed significant change in the expression of the *COX* and *LOX* genes in the surviving fish. Remarkably, the PGE_2_ levels were significantly elevated in fish from all treatment groups following the *S. iniae* challenge. Bacterial lipossacharides are a known stimulant for PGE_2_ synthesis (58, 59), which can be induced by both Gram-postive and Gram-negative bacteria (60, 61). Though we only measured PGE_2_ levels, an impact on other eicosanoids, in particular on PGE_1_, in MUT-supplemented diets should not be ruled out. PGE_2_ is generally regarded as a pro-inflammatory mediator that facilitates the formation of inflammatory cytokines (62). Recent studies have also highlighted an anti-inflammatory action of PGE_2_, which was shown to downregulate 5-LOX and upregulate 15-LOX, promoting the formation of anti-inflammatory lipoxins (63, 64). Thus, PGE_2_ may exhibit both pro-inflammatory and anti-inflammatory actions. The expression of COX-2 and the subsequent elevation in PGE_2_ production were documented to be associated with bacterial infections (33, 65, 66). Since all treatment groups were challenged with bacteria, the occurrence of elevated PGE_2_ levels was expected, though surprisingly, there was no apparent treatment effect. Only in the 15% WT-supplemented group, which provided the highest ARA, was a substantial increase in COX-2 gene expression measured in the same groups and correlated with the significant increase in PGE_2_. Cyclooxygenases are known to have a higher affinity to ARA as compared to DGLA, so higher PGE_2_ levels were expected in the ARA-supplemented groups, yet levels were similar among all treatment groups. It should be noted that the PGE_2_ assay kit used in this study exhibits up to 70% cross-binding with PGE_1_, which is the product of COX enzyme activity on DGLA. Thus, it is possible that the similar levels obtained were due to cross-reactivity. Since the post-challenge ARA levels decreased in all treatment groups, it can be assumed that this fatty acid was metabolized, including by COX enzymes, to substances that aided in the control of bacterial infection.

The elevated expression of the *LOX-1* and *LOX-2* genes, during the post-infection recovery period in the treatment groups, as compared to the control, is striking. *LOX-1* and *LOX-2* are homologous to the human *15-LOX* and *5-LOX*. 15-LOX is involved in the generation of the pro-resolving lipoxin A4 from ARA, suggesting that the higher expression aids in resolving the infection (6). LOX 15 products have anti-inflammatory and infection-resolving properties (67); thus, its expression at 1 week post-challenge may indicate a disease recovery stage. Despite the ongoing emphasis on the pro-inflammatory effects of ARA-derived eicosanoids, it is important to note the action of 15-LOX on ARA, producing the anti-inflammatory lipoxins A4 and pro-resolving 15-HETE (68). Previous studies have also demonstrated the effective role of leukotriene (LTB4) (product of 5-LOX) in antimicrobial defense involving the resolution of infections with various bacterial pathogens (69–72). The production of LTB4 is associated with processes such as an increase in vascular permeability, the release of lysosomal enzymes, and the generation of ROS and inflammatory cytokines, including TNF and different interleukins (73). These mentioned effects of leukotriene production correlated very well with the results of the gene expression patterns we obtained for lysozyme and inflammatory cytokines. Furthermore, leukotrienes also actively influence various effector functions of the innate immune response such as microbial phagocytosis and leukocyte accumulation (74). As emphasized in the review by Innes and Calder (6), it is evident that the interactions between different groups of PUFA, omega-3, which are part of the basic fish diet, and omega-6 PUFA, which were supplemented in this trial, and their lipid mediators in the context of inflammation are complex and still not properly understood. The results of the present study suggest gene upregulation, which may have prepared the fish better for a state of infection and allowed a quicker and more efficient response, such as the upregulated NF-κB, and thus better survival following challenge. After infection, gene expression acted for a more effective recovery, such as the upregulation of LOX genes.

The *S. inaie* challenge appeared to upregulate expression of IL1β in all treatments, but most prominently in the WT-supplemented diets. The upregulation of IL1β was expected as it is a cytokine mediating the inflammatory response toward endotoxin and liposaccharides (75, 76). The activation of TLR22 is generally linked with rapid activation of the innate imune system, and the expression of TNFα leads to macrophage activation and bacterial killing (77, 78). The lower expression of these genes at the time of challenge, as seen in the treatment groups, appeared to have benefited the fish in terms of resistance to the bacterial challenge. The explanation is not clear, but it may suggest that the fish were in a better physiological state in terms of balanced immune function and lower expression of immunostimulatory genes, when these were not needed. It should be noted that other components of algal cells, such as lipids, pigments and carbohydrates, may also play a definite role in modulating immune function and survival. These effects have been demonstrated in earlier studies that reported enhanced stress tolerance in fish when fed with ARA-rich TAG and β-carotene derived from *L. incisa* (11). Similar results of enhanced survival and resistance to acute stress were observed in guppy fry fed with *L. incisa* residue supplemented through artemia enrichment (14).

In this work, we focused on the potent effects of major n-6 LC-PUFA in experimental microalgae-supplemented diets, which appear to substantially affect immune function and infection resistance. It should be noted that microalgal biomass provides additional components that likely affected the immune and health status of treated fish, yet since the same alga was used in both cases, the main difference was in the *n*-6 LC-PUFA, WT, containing ARA, and the MUT, containing DGLA. Microalgae are a rich resource of functional food ingredients, encompassing health-promoting carotenoids, antioxidants, vitamins, essential aminoacids, and immunomodulating polysaccharides. Though we focused on the effects related to *n*-6 LC-PUFA application, more investigations should be performed in order to better understand the consequences of *L. incisa*-supplementation on the enhanced resistance to bacterial infection and immunomodulation in zebrafish. An important outcome of this work is that not only the balance between *n*-3 and *n*-6 LC-PUFA is important in sustaining the inflammatory homeostasis, but elevation in the dietary provision of *n*-6 LC-PUFA via microalgae-enriched diet exerts an apparent immunomodulatory effect in zebrafish and thus can be suggested as a potential health-promoting fish feed supplement.

## Author contributions

DZ, IK-G, and SN planned and carried out the experiments and the resulting interpretation. The EIA assay was carried out by GC. All authors contributed to the writing and editing of the manuscript.

### Conflict of interest statement

The authors declare that the research was conducted in the absence of any commercial or financial relationships that could be construed as a potential conflict of interest.
