# Supplementary material for: Dietary Supplementation With ω6 LC-PUFA-Rich Algae Modulates Zebrafish Immune Function and Improves Resistance to Streptococcal Infection
Source: Front Immunol. 2018 Sep 6;9:1960. doi: 10.3389/fimmu.2018.01960 (PMC6135890; doi:10.3389/fimmu.2018.01960)
Supplement: Supplementary file 1 [file Data_Sheet_1.pdf]

Supplementary Material

Dietary supplementation with ω6 LC-PUFA-rich algae modulates zebrafish immune function and improves resistance to Streptococcal infection

Sagar Nayak, Inna Khozin-Goldberg*, Guy Cohen, Dina Zilberg*

* Correspondence:
Dina Zilberg
dzilberg@bgu.ac.il
Inna Khozin-Goldberg
Khozin@bgu.ac.il

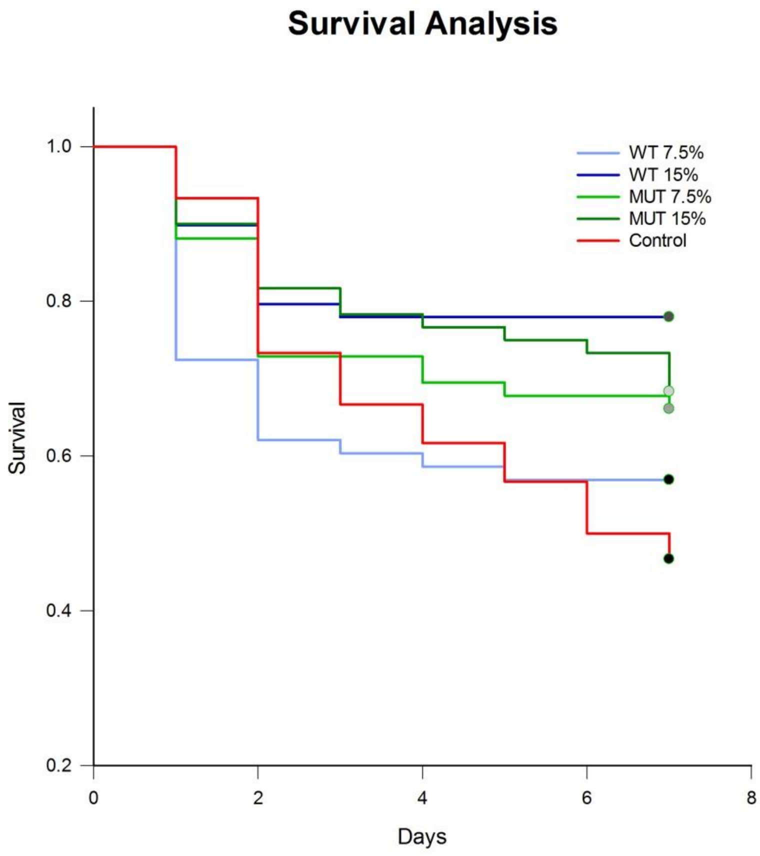

Supplementary Figure 1. Kaplan-Meir plot of survival following *S. iniae* challenge in zebrafish fed with different experimental diets (log rank statistic for the survival curves indicate of significant difference, p = 0.012); (n = 4, 20 fish/tank).

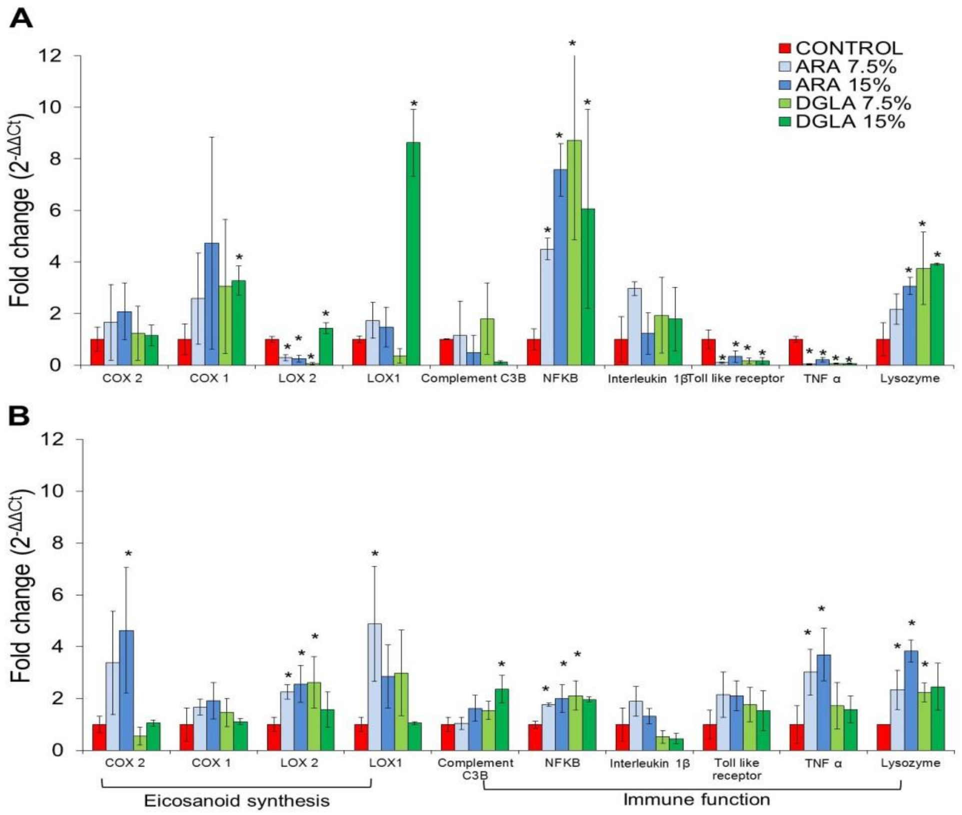

Supplementary Figure 2. Real-time PCR quantification of mRNA expression of genes related to eicosanoid synthesis and immune function in zebrafish kidneys. Plots represent average fold change in expression of mRNA normalized to expression of control unsupplemented group (n = 4 biological replicates). **A**) Expression of genes after four weeks of dietary supplementation. **B**) Expression of genes at one week after *S. iniae* challenge. * denotes significant difference from the control.

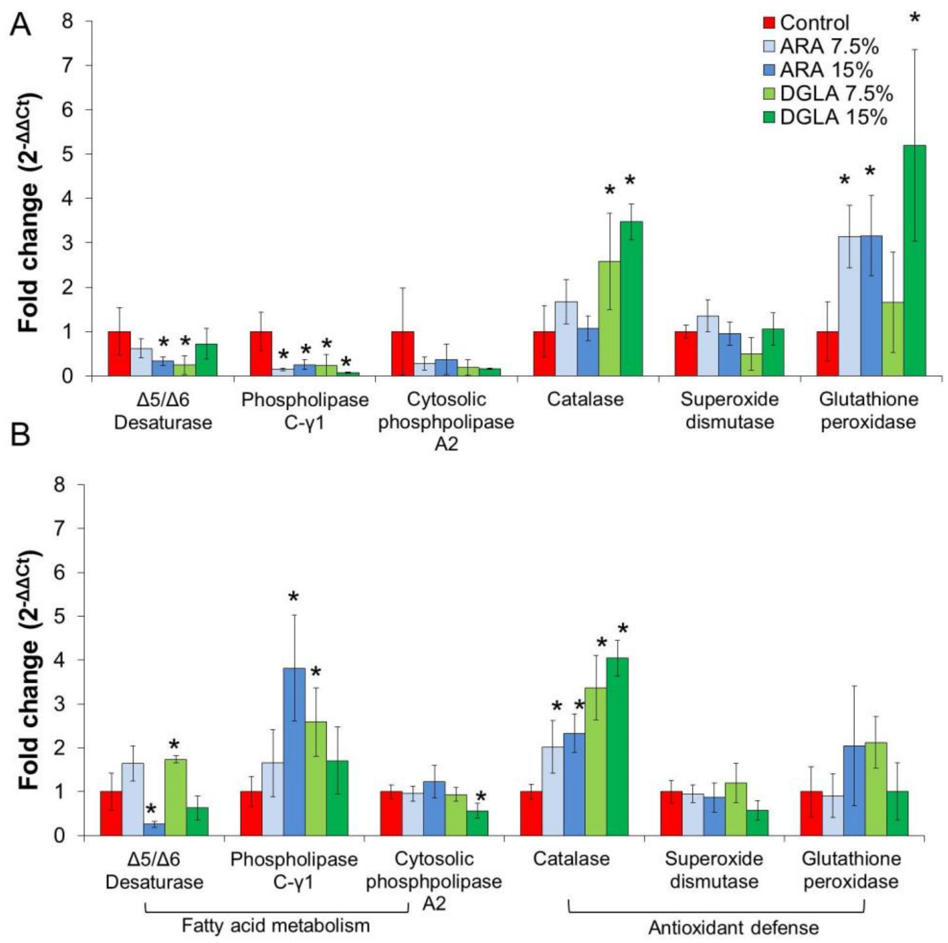

Supplementary Figure 3. Real-time PCR quantification of mRNA expression of genes related to fatty acid metabolism and antioxidant defenses in zebrafish liver. Plots represent average fold change in expression of mRNA normalized to expression of control un-supplemented group (n = 4 biological replicates). A) Expression of genes after four weeks of dietary supplementation. B) Expression of genes at one week after *S. iniae* challenge. * denotes significant difference from the control.

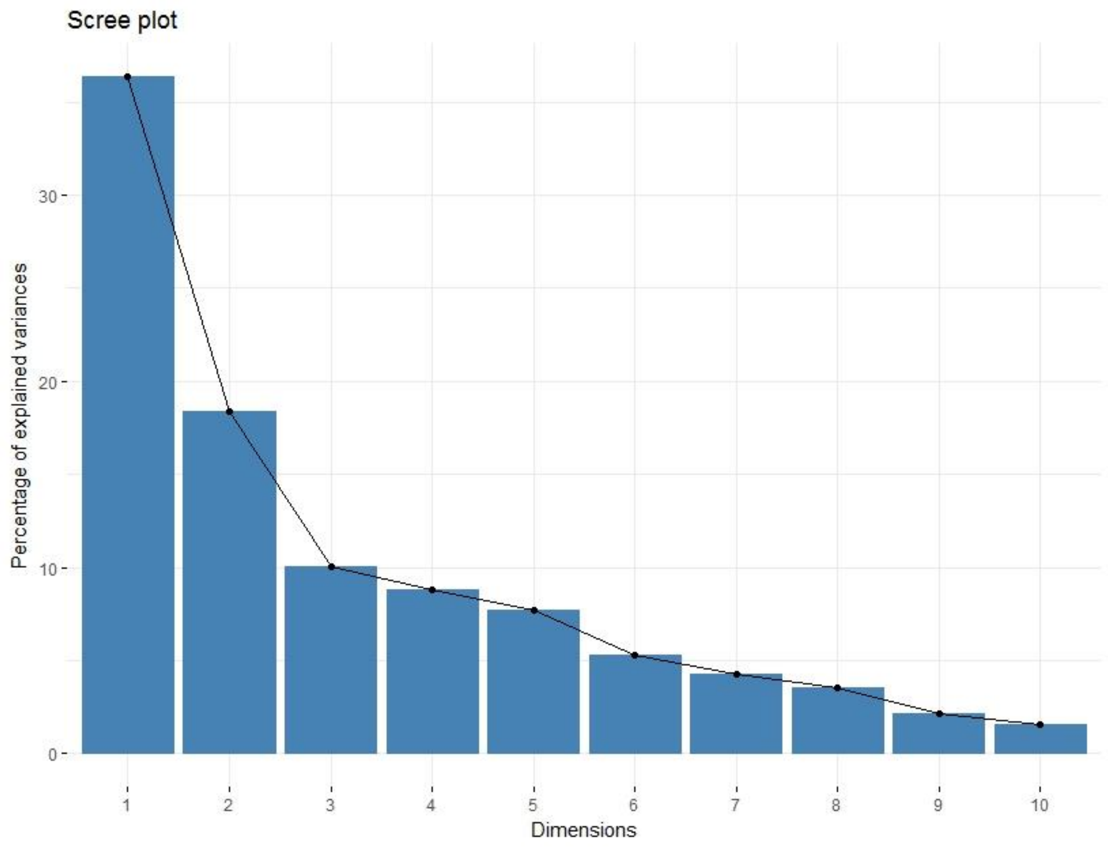

Supplementary Figure 4. Scree plot obtained from principal component analysis based on eigenvalues. Values in plot represent the percentage of variance explained by each dimension or component.
